# Characterizing Help-Seeking Searches for Substance Use Treatment From Google Trends and Assessing Their Use for Infoveillance: Longitudinal Descriptive and Validation Statistical Analysis

**DOI:** 10.2196/41527

**Published:** 2022-12-01

**Authors:** Thomas Patton, Daniela Abramovitz, Derek Johnson, Eric Leas, Alicia Nobles, Theodore Caputi, John Ayers, Steffanie Strathdee, Annick Bórquez

**Affiliations:** 1 Division of Infectious Diseases and Global Public Health University of California San Diego La Jolla, CA United States; 2 Department of Medicine University of California San Diego La Jolla, CA United States; 3 Medecins Sans Frontieres Genève Switzerland; 4 Herbert Wertheim School of Public Health & Human Longevity Science University of California San Diego La Jolla, CA United States; 5 Department of Economics Massachusetts Institute of Technology Cambridge, MA United States

**Keywords:** internet, search, help-seeking, substance use treatment, surveillance, infoveillance, google trends

## Abstract

**Background:**

There is no recognized gold standard method for estimating the number of individuals with substance use disorders (SUDs) seeking help within a given geographical area. This presents a challenge to policy makers in the effective deployment of resources for the treatment of SUDs. Internet search queries related to help seeking for SUDs using Google Trends may represent a low-cost, real-time, and data-driven infoveillance tool to address this shortfall in information.

**Objective:**

This paper assesses the feasibility of using search query data related to help seeking for SUDs as an indicator of unmet treatment needs, demand for treatment, and predictor of the health harms related to unmet treatment needs. We explore a continuum of hypotheses to account for different outcomes that might be expected to occur depending on the demand for treatment relative to the system capacity and the timing of help seeking in relation to trajectories of substance use and behavior change.

**Methods:**

We used negative binomial regression models to examine temporal trends in the annual SUD help-seeking internet search queries from Google Trends by US state for cocaine, methamphetamine, opioids, cannabis, and alcohol from 2010 to 2020. To validate the value of these data for surveillance purposes, we then used negative binomial regression models to investigate the relationship between SUD help-seeking searches and state-level outcomes across the continuum of care (including lack of care). We started by looking at associations with self-reported treatment need using data from the National Survey on Drug Use and Health, a national survey of the US general population. Next, we explored associations with treatment admission rates from the Treatment Episode Data Set, a national data system on SUD treatment facilities. Finally, we studied associations with state-level rates of people experiencing and dying from an opioid overdose, using data from the Agency for Healthcare Research and Quality and the CDC WONDER database.

**Results:**

Statistically significant differences in help-seeking searches were observed over time between 2010 and 2020 (based on *P*<.05 for the corresponding Wald tests). We were able to identify outlier states for each drug over time (eg, West Virginia for both opioids and methamphetamine), indicating significantly higher help-seeking behaviors compared to national trends. Results from our validation analyses across different outcomes showed positive, statistically significant associations for the models relating to treatment need for alcohol use, treatment admissions for opioid and methamphetamine use, emergency department visits related to opioid use, and opioid overdose mortality data (based on regression coefficients having *P≤*.05).

**Conclusions:**

This study demonstrates the clear potential for using internet search queries from Google Trends as an infoveillance tool to predict the demand for substance use treatment spatially and temporally, especially for opioid use disorders.

## Introduction

Understanding help-seeking behavior for substance use treatment is critical for the effective deployment of resources. This presents a challenge to researchers and policy makers because there is no recognized gold standard method for estimating the number of individuals with substance use disorders (SUD) within a given geographical area [1]. A standard approach involves asking a sample of the general population questions about their substance use, either through surveys or in-depth interviews [2]. Unfortunately, these sources are subject to well-known limitations, such as low participation rates, lag time between data collection and published results, and data availability [3]. Additionally, survey scale-up is not always feasible given both costs and concerns about participant burden [4].

Indirect estimation approaches have been used, including capture-recapture [5], multiplier [6], and data triangulation methods [7], but these methods are also subject to limitations, either in the form of impractical data requirements or the potential for bias [7,8]. Finally, efforts have been made to collect data on drug-related harms, such as overdose statistics, although time-lags in their dissemination have meant that these initiatives have struggled to keep pace with the rapidly evolving opioid epidemic in the United States [9]. In view of this methodological backdrop, there has been limited scope to develop real-time surveillance mechanisms to guide policy responses.

A promising development has emerged in the use of internet search queries related to substance use [10]. One of the main benefits of this approach to substance use surveillance is that the data are publicly accessible and can be easily obtained in real time [11]. There is a growing body of research exploring the use of internet search data for the surveillance of substance use trends. A study in 2018 found strong and significant correlations between Google search data for novel psychoactive drugs and annual drug use prevalence, collected in a nationally representative US sample [10]. Two studies explored the relationship between drug-related internet search queries and opioid-related emergency department (ED) visits in the United States, and both demonstrated the predictive potential of internet search data [12,13]. Three further studies found strong associations between drug-related internet search queries and opioid-related overdose deaths at the national, state, and county levels [14-16]. Elsewhere, studies have demonstrated the potential use of opioid-related data from social media platforms, including Twitter and Reddit, to inform surveillance efforts [17-19].

While the previous literature has focused on the use of internet data as a proxy for real-time data on opioid-related health harms, this study provides new insights into the use of internet search data to explore SUD help seeking for a broad range of substances, including cocaine, methamphetamine, opioids, cannabis, and alcohol, and validate these against observed SUD indicators. These substances were chosen because they are the 5 most common types of substance that people are admitted to treatment for in the United States [20]. By validating surveillance of SUD help seeking as a methodological tool, it is our hope that key stakeholders, including local health departments, harm reduction organizations, and researchers, will be better able to proactively respond to need [21]. We first described help-seeking searches for cocaine, methamphetamine, opioids, cannabis, and alcohol at national and state level from 2010 to 2020 in the United States and characterized heterogeneity in these outcomes between states.

We sought to determine the feasibility of using search query data as a low-cost and real-time indicator of unmet treatment need, demand for treatment, and a predictor of the health harms related to unmet treatment needs. The exploratory nature of this study warrants a continuum of hypotheses to account for different outcomes that might be expected to occur depending on the relative demand versus capacity for treatment. If there is sufficient treatment capacity, one would expect to see a strong, positive association between help-seeking searches and treatment admissions. However, given the limited capacity for SUD treatment in the United States, it was important to consider additional hypotheses; if there is excess demand for treatment, we would expect to see a weaker association between help-seeking searches and treatment admissions but a stronger association with unmet treatment need and drug-related health harms. In addition, it is also key to acknowledge that treatment seeking for SUD is a complex process that involves moving, often nonlinearly, through different stages of behavior change [22]. Therefore, considering several outcomes also allows us to reflect the different situations that individuals with SUD, or those around them and trying to help, might be experiencing.

We tested 3 hypotheses, the first of which posits that treatment-seeking searches are positively associated with unmet treatment needs, as searching for help indicates that the person is struggling with their substance use and is considering treatment as an option but has not yet received help (ie, contemplation). Next, we tested the hypothesis that treatment-seeking searches are positively associated with treatment admissions, as searching for help is an indicator that the person is actively seeking to engage (ie, preparation/action) [22,23]. Finally, we tested the hypothesis that treatment-seeking searches are positively associated with nonfatal and fatal overdose, as expressing a treatment need often occurs in the latter stages of SUD, when symptoms are more severe, leading to an increased risk of overdose and death (ie, contemplation, preparation, or relapse) [24]. We identified relevant variables across different state-level data sources to validate the models for each of these outcomes and determine whether internet searches for substance use help seeking can be used to enhance SUD treatment need surveillance and treatment linkage efforts.

## Methods

### Extraction of Google Search Query Data

We obtained Google queries in November 2020 originating from the United States that included the terms “quit,” “stop,” “rehab(s),” “rehabilitation,” “treatment(s),” “help,” or “detox” in combination with (A) alcohol (“alcohol,” “alcoholic,” or “alcoholism”), (B) cannabis (“cannabis” or “marijuana”), (C) cocaine (“cocaine”), (D) methamphetamine (“methamphetamine” or “meth”), or (E) opioids (“opioid(s),” “heroin,” “fentanyl,” “oxycontin,” “oxycodone,” “codeine,” “hydrocodone,” “morphine”) from January 1, 2010, to November 1, 2020. For example, “Where can I get help for alcoholism” would be included in the alcohol help-seeking search category. These searches were specified without quotation marks, and the data were obtained by selecting the “search terms” option, as opposed to the “topics” option. The search terms for our drugs of interest corresponded to the standard dictionary term for each (eg, methamphetamine), alongside other commonly used terms if relevant (eg, meth) based on the authors’ expertise in SUD and others’ contributions in this field [12,13,16]. For opioids, we also included names of most frequently used street drugs (ie, heroin, fentanyl) and prescription drugs with their brand name if very commonly used (eg, oxycodone and OxyContin). For alcohol, we also included “alcoholic” and alcoholism,” as these are part of the mainstream English lexicon used to describe alcohol use disorders. Despite the extensive range of slang terms used to describe drugs [25], these were not included, given that slang is ever evolving, its linguist survival is often short-lived, and it is typically context specific and limited in use within specific social settings [26]. Given our focus on treatment seeking (ie, a formal context), our broad geographical scale (ie, all US states), and our extended time scale (10 years), we opted to limit our search to the most standard terms to allow for consistency over time and space. The search query data were obtained for each calendar year between 2010 and 2020 from Google Trends using the Google Application Programming Interface (API) Client library in Python [27]. Trends in Google queries were measured in query fractions (QFs), which estimate the number of searches that mention substance-specific keywords, in combination with the help-seeking keywords, in the time frame and geography divided by the total number of searches in the same time frame and geography and expressed as a rate per 1 million searches. This approach facilitates comparability by adjusting for changes in Google usage over time, as well as differences across states and substance types.

### Statistical Analysis of Google Search Query Data

Negative binomial regression models were fitted to the QF data to make inferences regarding the significance of temporal changes in help-seeking queries. Negative binomial regression is commonly used to analyze count and rate data exhibiting over-dispersion (ie, variance greater than the mean) [28]. The QF data in this study were found to be overdispersed, as shown in Table 1; therefore, the negative binomial model was chosen to analyze these data. The model specifications included a main fixed effect for year (ie, 2010 through 2020). Random effects were included for intercept terms to account for differences between states at the beginning of the study and for correlations between data points collected in the same states over different years. Moreover, autocorrelated error terms were specified to account for correlations in the data between successive time points. A Wald test was performed to confirm whether the variable “year” was statistically significant for each of the models [29]. We calculated Gini coefficients [30] to quantify the dispersion of help-seeking queries across states for each substance and each year.

**Table 1:** Descriptive statistics from 2010 to 2020 for annual search query fractions (QFs) by substance type^a^.

Statistic	Alcohol	Cannabis	Cocaine	Meth^b^	Opioids	Composite^c^
Mean	27.3	8.4	2.5	4.2	8.0	12.7
Median	25.6	7.4	2.2	3.7	7.2	12.8
Minimum	10.3	2.7	1.0	0.8	2.4	9.1
Maximum	70.7	48.0	9.8	35.0	37.7	16.6
SD	7.2	3.7	1.0	3.3	3.5	1.8
IQR	7.3	3.2	0.7	2.9	2.7	2.8

^a^Query fractions (QFs) refer to queries per every 1 million total Google searches.

^b^Meth: methamphetamine.

^c^Variable estimated by combining QF statistics for opioid, methamphetamine, and cocaine use treatment seeking.

### Validation of Google Search Queries as Indicators of Unmet Treatment Needs for Substance Use (Hypothesis 1)

First, an analysis exploring the number of people needing but not receiving treatment at a specialty facility for SUD in the past year was conducted using data from the National Survey on Drug Use and Health (NSDUH) for the years 2016 to 2019. The NSDUH is an annual state-level representative survey of the civilian, noninstitutionalized population aged 12 or older and is publicly accessible from the website of the Substance Abuse and Mental Health Services Administration (SAMHSA) [31]. To produce state-level estimates for variables collected in this survey (rounded to the nearest thousand), the Research Triangle Institute conducted an analysis of the sample data for each year using survey-weighted hierarchical Bayes methods [32]. The NSDUH separately enquires about needing but not receiving treatment at a specialty facility for alcohol and illicit drug use in the past year. Therefore, a negative binomial model was utilized to regress NSDUH estimates specific to alcohol use on the variables of alcohol QF and year, and a second analysis was conducted exploring illicit drug use, also using a negative binomial regression model. The main fixed effects included in the second model were a composite QF statistic, estimated by combining QF statistics for opioid, methamphetamine, and cocaine use unmet treatment need and the year corresponding to the data points. For both sets of analyses involving NSDUH data, random effects were specified for intercept terms and the natural logarithm of states’ population estimates from the US Census Bureau as offset terms [33], which reflect the number of times the event could have potentially occurred. Additionally, interactions between the main fixed effects were assessed to infer if and how the association between alcohol QF and treatment need as well as the association between the composite illicit drug QF and treatment need varied across the years.

### Validation of Google Search Queries as Indicators of Treatment Seeking for Substance Use Disorders (Hypothesis 2)

We investigated whether there was a positive association between treatment-seeking searches and the receipt of treatments for SUD. For the latter, data were obtained from the Treatment Episode Data Set: Admissions (TEDS-A) data sets (years 2012 to 2018) on the number of admissions to substance use facilities by primary substance for which treatment was sought [34]. Observations from these data were only selected for admissions involving individual referrals for treatment (ie, excluding mandated treatment visits). Negative binomial regression models were fitted to the admissions data with separate analyses for the different types of substance use (alcohol, cannabis, opioids, cocaine, and methamphetamine). In each model, the year and corresponding help-seeking QF variable (ie, substance-specific) were included as main fixed effects, along with intercepts for the states as random effects and the natural logarithm of states’ population estimates from the US Census Bureau as offset terms [33]. Additionally, interactions between the main fixed effects variables (ie, help-seeking QF and year) were assessed to infer if and how the association between treatment-seeking searches and admissions varied across the years.

### Validation Of Google Search Queries as Predictors of Health Harms Related to Unmet Treatment Need (Hypothesis 3)

We investigated whether help-seeking searches were positively associated with nonfatal and fatal opioid overdose. Accordingly, data on the rate of ED encounters associated with opioid use per 100,000 people (mostly corresponding to nonfatal overdoses) were obtained from the Agency for Healthcare Quality and Research (AHRQ) [35], and data on the number of opioid-related overdose deaths were obtained from the CDC WONDER database (using the criteria set out in previous research [36]). For these analyses, we regressed state-specific opioid hospitalization rates and mortality count data, respectively, on the variables opioid QF and the year corresponding to the data points, using a negative binomial specification. Once again, random intercepts were included to account for correlations between repeated observations within states. The analysis of fatal opioid overdose data included an offset term corresponding to the log of the state-level population. This approach was not taken for the AHRQ data, as these data were obtained in the form of rates, rather than count data. The interaction terms between the main fixed effects (ie, opioid QF and year) were also assessed.

All analyses were conducted using R software (version 4.1.0.), and the negative binomial models were fitted using the glmmTMB package [28].

### Ethical Considerations

Ethical review was not required because the study relied on public, aggregated, and deidentified data. Given that this study relied on the use of secondary deidentified data (numbers were aggregated to the state level), the Institutional Review Board of the University of California San Diego determined that an ethics review was not required (Project #200332XX).

## Results

### Descriptive Analysis of Google Search Query Data for SUD Help Seeking

**Figure 1:**
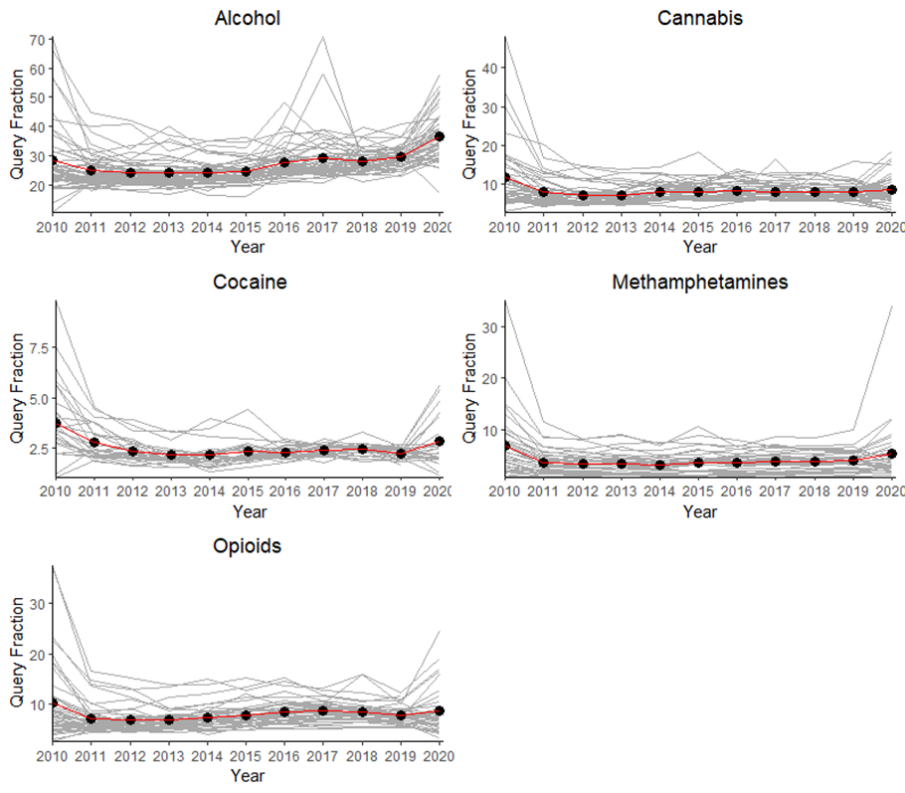
Average help-seeking trends for substance use. Gray lines represent state-specific trends while black dots represent the mean estimates for states* with data across all time points. *Number of states (plus the District of Columbia) with nonmissing query data by substance: Alcohol=51, Opioid=41, Cannabis=44, Methamphetamine=32, Cocaine=25. Number of data points by substance: Alcohol=561, Opioid=461, Cannabis=484, Methamphetamine=382, Cocaine=285.

Figure 1 shows that QF values were highest on average in 2010 for all substances, except in the case of alcohol, where it was the second highest year for searching, and the highest levels were observed in 2020. Help-seeking searches were lowest in 2012 in the case of opioids, in 2013 in the case of alcohol and cannabis, and 2014 in the case of cocaine and methamphetamine. Figure 1 also shows the varying levels of completeness in the search query data across the different types of SUD. Missing data points can occur in cases involving very low search volumes [37].

### Statistical Analysis of Time and Geographic Trends in Google Search Query Data for SUD Help Seeking

The negative binomial regression analyses of QF data (results shown in Table S1 in Multimedia Appendix 1) showed that the variable “year” was statistically significant for all substance types based on the resulting Wald tests (*P*<.001). This indicates that there were important variations in help-seeking searches between the various years. Pairwise comparisons tests for significant differences in the help-seeking search counts over consecutive years were performed by applying Bonferroni corrections to the outputs of the negative binomial regression analyses (results shown in Table S2 in Multimedia Appendix 1). All substances showed significant decreases from 2010 to 2011 (alcohol: 10%, cannabis: 21%, cocaine: 25%, methamphetamine: 43%, opioids: 26%). Aside from this, significant differences across consecutive years were found for alcohol (13% increase from 2015 to 2016 and 21% increase from 2019 to 2020) and methamphetamine (23% increase from 2019 to 2020).

**Figure 2:**
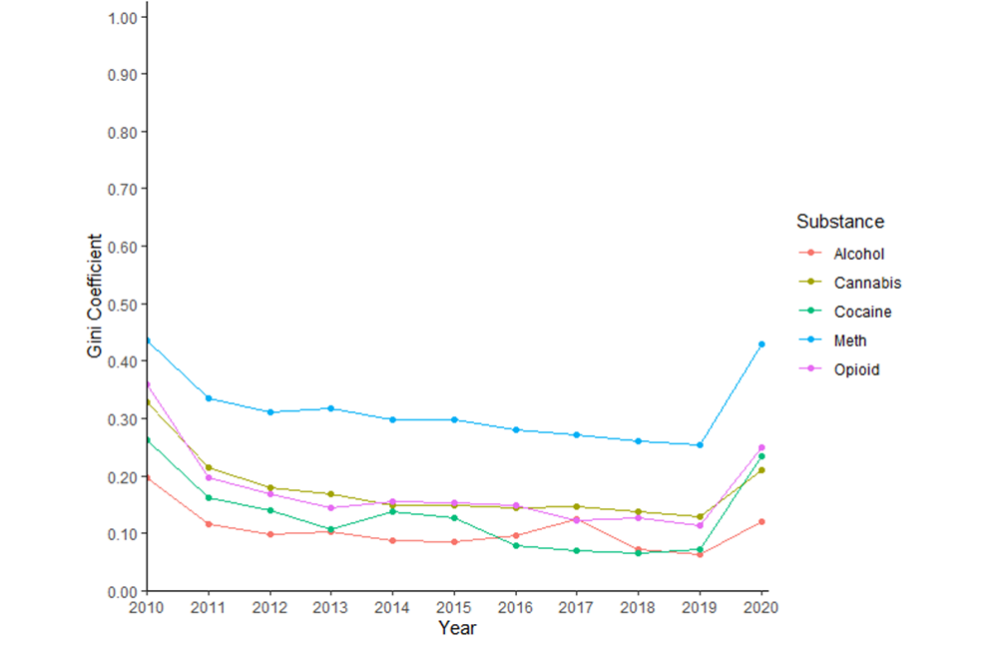
Gini coefficient estimates from query fractions (QF) variables across substances and years.

**Figure 3:**
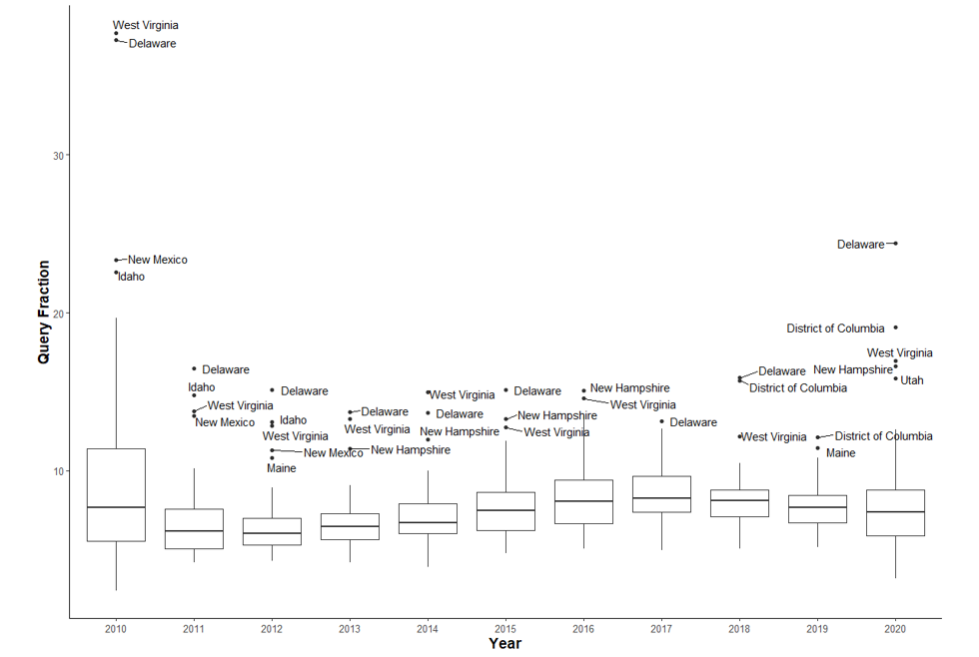
Box and whisker plot of help-seeking searches for opioid use.

Table 1 provides descriptive statistics for each of the QF variables to facilitate the interpretation of all subsequent regression analyses where these were employed as independent variables. Inequalities in help-seeking searches between states, as measured by Gini coefficients, were highest for methamphetamine across all years (Figure 2). Inequalities were lowest for alcohol for all years except those between 2016 and 2018, when cocaine was the lowest. A consistent trend observed across all substances was that inequalities were highest in 2010 and then reduced over time before sharply increasing again in 2020. These results can also be further understood by looking at the box and whisker plots, which show the spread of data points across states by substance type and year (see Figure 3 for opioid use and Figure S1 in Multimedia Appendix 1 for other substances).

### Validation of Google Search Queries for SUD Help Seeking as Indicators of Unmet Treatment Need for SUD (Hypothesis 1)

The analysis of NSDUH data showed a statistically significant (*P*=.004) association between QF and the number of people needing but not receiving treatment for alcohol use at a specialty facility (rate ratio changes are shown in Table 2, and regression outputs can be found in Tables S3 and S4 in Multimedia Appendix 1). The estimates were not significantly different from 0 in the case of illicit drug use (*P*=.26). The coefficient estimates for alcohol use and illicit drug use confirmed our expectation of a positive association between the QF variables and the number of people needing but not receiving treatment for their substance use. After adjusting for variations across years, both the analyses for alcohol use and illicit drug use showed that a 1-unit increase (ie, 1 additional search per million searches) in the composite QF variable approximately corresponded to a 1% increase in the expected rate of people needing but not receiving treatment for alcohol use and illicit drug use, respectively. Neither analysis showed statistically significant interactions between QF variables and the variable “year.”

Predictions were made for the model analyzing the number of individuals needing but not receiving treatment for alcohol use on account of the significant QF finding. No evaluation of predictive performance was conducted for the illicit drug use model because its association with the QF variable was not statistically significant. Comparisons between the predicted and observed rates of people needing but not receiving treatment for alcohol use are presented in Figure S2 in Multimedia Appendix 1. The predictive performance was also quantified by calculating root mean squared errors (RMSE), comparing observed and predicted rates. The mean RMSE was 697, which is low when compared to the mean rate of 5284 per 100,000 people needing but not receiving treatment for alcohol use. The resulting scatter index of 13%, which is calculated by dividing the mean RMSE by the mean rate and then multiplied by 100, suggests a reasonable predictive performance based on previously used benchmarks [38]. Predictive performance was also examined over time and across states/territories (Tables S5-S6 in Multimedia Appendix 1). It was shown to be best in 2019 for the states of Idaho, Virginia, Michigan, New York, and Kansas and worse in 2018 for the District of Columbia, Colorado, Oregon, Montana, Vermont, compared to other years and states, respectively.

**Table 2:** Estimates of the rate ratio change in number of people needing but not receiving treatment (NSDUH) associated with a one unit increase in the query fractions (QFs) variable.

Variable	Estimate	95% CI	*P* value
Illicit drug use	1.01	0.99-1.03	.26
Alcohol use	1.01	1.00-1.01	.004

### Validation of Google Search Queries for SUD Help Seeking as Indicators of Treatment Seeking for SUD (Hypothesis 2)

The analysis of TEDS-A data showed that the association between help-seeking searches and the receipt of treatments for SUD varied by substance type (rate ratios are shown in Table 3, and regression outputs can be found in Tables S7 to S11 in Multimedia Appendix 1). Statistically significant and positive associations between these variables were found for methamphetamine use (*P*<.001). Interactions between the methamphetamine QF and the year variable were nonsignificant and thus ruled out (*P*=.88 for Type III Wald test). The model outputs showed that a 1-unit increase in the methamphetamine QF variable approximately corresponded to a 26% increase in the expected rate of treatment episodes. Statistically significant and positive associations were also found for opioids (*P*<.001). Interactions between the opioid QF and the year variable were nonsignificant and thus ruled out (*P*=.26 for Type III Wald test). The outputs from the model showed that a 1-unit increase in the opioid QF variable approximately corresponded to a 12% increase in the expected rate of treatment episodes.

In the case of cannabis, the association was also positive and slightly above a 5% statistical significance criterion (*P*=.07). Although the data did not allow strong inferences to be drawn from the analysis of cannabis data, the outputs from the model showed that a 1-unit increase in the cannabis QF variable approximately corresponded to a 3% increase in the expected rate of treatment episodes. Findings for the analyses of alcohol and cocaine use showed both nonsignificant association between treatment-seeking searches and the receipt of treatments (*P*=.92 for alcohol use and *P*=.22 for cocaine use). Neither model exhibited significant interactions between the QF and the year variable (*P*=.23 for alcohol use and *P*=.88 for cocaine use for Type III Wald test for the models of treatment).

**Table 3:** Estimates of the rate ratio change in number of individual treatment referrals associated with a 1 unit increase in the query fractions (QFs) variable.

Variable	Estimate	95% CI	*P* value
Alcohol QF^a^	1.00	0.99-1.01	.92
Cannabis QF	1.03	1.00-1.07	.07
Cocaine QF	0.90	0.76-1.07	.22
Meth QF	1.26	1.17-1.36	<.001
Opioid QF	1.12	1.07-1.17	<.001

^a^QF: query fraction.

Predictions were made for the models analyzing admissions to treatment for methamphetamine and opioid use. Comparisons between the predicted and observed rates of admission to treatment are presented in Figures S3-S4 in Multimedia Appendix 1. The predictive performance of the models for opioid and methamphetamine use was also quantified by calculating RMSE. The mean RMSE for methamphetamine was 11.7, which indicates a poor predictive performance, given that the mean admission rate was 15.2 per 100,000 people. The predictive performance was also shown to be weak for opioids based on comparisons between the mean RMSE (77.9) and the mean admission rate (102.4 per 100,000 people). Predictive performance was also examined over time and across states (Tables S12 and S13 in Multimedia Appendix 1). For both substances, predictive performance was found to be best in 2011 compared to other years for both substances, and in the states of Indiana, Texas, New York, and Michigan for methamphetamine use and in Illinois, Ohio, Missouri, Utah for opioid use. It was the worst in 2018 for both substances, compared to other years, and generally worse among states with higher admission rates.

### Validation Of Google Search Queries for SUD Help Seeking as Predictors of Health Harms Related to Unmet Treatment Need (Hypothesis 3)

The analysis investigating the relationship between treatment-seeking searches for opioid use and opioid-related emergency department visits using AHRQ data showed a positive and statistically significant association (*P*<.001, see Tables S14-S15 in Multimedia Appendix 1). However, statistically significant interactions between the opioid QF and the variable year were identified, indicating that the relationship was not stable over time (Type III Wald test *P*=.005). An evaluation of the simple main effects of the opioid QF by year showed a decreasing trend over time (Table S14 in Multimedia Appendix 1). In 2011, a 1-unit increase in the opioid QF variables was associated with a 6% increase in the expected rate of opioid-related emergency department visits, but by 2018, there was a nonsignificant association between these variables. No evaluation of predictive performance was conducted for this model, as the association with the QF variable was found to vary over time.

The analysis investigating the relationship between treatment-seeking searches for opioid use and opioid overdose mortality counts using CDC WONDER data showed a positive and statistically significant association (*P*<.001, see Table 4 and Table S16 in Multimedia Appendix 1). Interactions between the opioid QF and the year variable were nonsignificant and thus ruled out (Type III Wald test *P*=.11). The outputs from the model showed that a 1-unit increase in the opioid QF variable corresponded to a 11% increase in the expected overdose mortality count (Table 4). The predictive performance for the model was determined by estimating the RMSE. The relative difference between the mean RMSE (4.3) and the mean admission rate per 100,000 people (12.2) indicated a better predictive performance, on average, when compared to the models predicting treatment admission rates. Figure S5 in Multimedia Appendix 1 illustrates the differences between predicted and observed mortality rates across states. Predictive performance was best in 2013 compared to other years (Table S17 in Multimedia Appendix 1) and in the states of New York, Florida, Virginia, and Wisconsin compared to other states (Table S18 in Multimedia Appendix 1). It was worst in 2017 and in West Virginia, Ohio, Idaho, and Maryland, where opioid overdose mortality was very high (with the exception of Idaho).

**Table 4:** Estimates of the rate ratio change in number of overdose deaths associated with a 1-unit increase in the opioid query fractions (QFs) variable.

Variable	Estimates	95% CI	*P* value
Opioid QF^a^	1.11	1.09-1.14	<.001

^a^QF: query fraction.

## Discussion

### Principal Findings

To our knowledge, this is the first study to retrospectively describe spatial and temporal changes in substance use searches in the United States and rigorously investigate their association with outcomes along the continuum of care (and absence of care) for SUD. In the future, monitoring of Google search queries with validated metrics may allow the prospective identification of variations by substance and state indicating specific SUD treatment information and linkage needs in the population, providing useful near real-time insights to public health organizations developing and delivering campaigns for SUD treatment. Key stakeholders (local health departments, harm reduction organizations, etc) could then better allocate resources to target SUD treatment needs (eg, a digital intervention in real time) for each substance in specific states. For instance, between 2010 and 2020, West Virginia (methamphetamine and opioids), New Mexico (methamphetamine), Delaware (opioids), and Connecticut (cocaine) were repeatedly found to exhibit high levels of demand for information on SUD treatments that were potentially unmet.

Importantly, the positive and significant associations we identified between help-seeking searches for opioid and methamphetamine use and admissions to substance use treatment facilities suggests that, at least for these 2 substances, internet search data represents a valuable resource to assess treatment seeking. Interpreting the magnitude of these associations should be considered in the context of the baseline rate of treatment admissions and the overall population size for a given state. For instance, the implications of a 1-unit increase in the rate of help-seeking searches for methamphetamine use in California differs vastly from that in Virginia. The average rate of treatment admissions per 100,000 across all years was 37.43 for California and 1.12 for Virginia. Given that a 1-unit increase in the rate of help-seeking searches is associated with a 26% increase in treatment admissions, this corresponds to 9.73 additional admissions per 100,000 for the average rate in California and 0.29 additional admissions per 100,000 for the average rate in Virginia. In absolute terms, this equates to over 3800 additional admissions for California and only 5 additional admissions for Virginia.

Further analyses showed significant associations between help-seeking searches for opioid use and data on health harms related to unmet treatment need. These findings have implications for both surveillance and treatment, as they demonstrate the clear potential of search query monitoring to fill existing gaps and indicate that the internet likely represents a strategic platform to link people in need of treatment to services. This is especially important given that there are well-documented challenges in estimating the prevalence and incidence of SUD [5,8]. As such, leveraging internet search platforms could make health agencies more responsive to both information and treatment referral needs. This potential can be realized by developing a surveillance platform for real-time monitoring and linkage to services that can allow users to rapidly evaluate fluctuating patterns in SUD help seeking and implement strategic outreach. To realize this potential, search data need to be measured in terms of QFs to ensure that data points are comparable over time and across states. This approach was achieved in this study by extracting search data using the Google API Client library. It is important to highlight that this is not achieved when data are extracted directly through the Google Trends website but rather when data are normalized according to the selected time frame and geographical region [37].

### Limitations

Our study is not without limitations. Several states were missing search query data for SUD help-seeking behavior because Google Trends will only report search queries if they are above a minimum threshold. There was variation in the predictive performance of our models over time and across states. In particular, performance was lowest in states where rates of treatment admissions or overdose mortality rates were very high, which is expected when using RMSE as the performance indicator since it penalizes large errors. Using search data may be subject to selection bias, as not all people access the internet equally. Although some queries may reflect general curiosity rather than help seeking, it is well known that internet search trends mirror many health-related behaviors [39], and in the specific case of SUD, that of family members, partners, and friends trying to help their loved one [39]. Another potential confounding factor is the fact that the Google search algorithm is nonstatic. Search patterns change over time due to the thousands of decisions being made by Google’s programmers as the company strives to test and improve its search algorithm [40]. This could lead to temporal changes in the likelihood of individuals successfully finding treatment following an online search. As such, this phenomenon could distort the association between search trends and treatment admissions.

While our approach may overcome many of the ongoing limitations in substance use surveillance (ie, a lack of timely, substance specific, and publicly available data), the finest granularity of aggregate Google search data is limited to designated marketing areas [41], so it does not necessarily align with the jurisdictional level of public health departments. The approach taken in this paper also assumes that search queries are made using standard terms for SUD in the context of treatment seeking, which disregards instances where people might use slang terms. It is also possible that the predictive value of specific terms varies between states and over time. However, given the nonpunitive nature of online help seeking for SUD (as compared with that of purchasing or selling drugs), we expect this to be limited. It is important to recognize the potential limitations of using data on the number of treatment-seeking visits from TEDS-A. Given that these data are collected from facilities receiving public funding, the findings from analyses using this data potentially misrepresent associations for states with greater reliance on private funding or nonspecialty settings such as office-based outpatient treatment. Other potential confounding factors include geographical and temporal variations in the number of help-seeking queries in other languages, the proportion of queries coming from surrogate seekers [42], and the use of alternative search engines. In particular, including searches using Spanish terms would have a heterogeneous impact across states, and the relationship between searches and health outcomes might be different depending on the policies and interventions in place to facilitate healthcare access among non-English speakers and those who are undocumented [43,44]. This warrants a separate study focusing on Spanish language terms and SUD-related health outcomes among Hispanic individuals.

Importantly, the strength and significance of associations between searches and outcomes along the continuum of care varied depending on the substance and outcome, as well as between states and through time. This is expected, given that there have been heterogeneities in drug policies over time and across States. Between 2010 and 2020, cannabis was made legal in 19 states for medical use, in 8 states for recreational use, and in 3 states for medical use first and then later for recreational use [45]. Given that the impacts of legalization on the social acceptability of treatment-seeking behaviors are still poorly understood [46], it is difficult to surmise whether changes in the legal status of cannabis across states and over the duration of the study may have had a distorting impact on the results. Another key policy area is the state adoption of naloxone access laws (NAL), which increased rapidly from 2013 onward [47]. By 2020, all 50 states and the District of Columbia had some form of NAL in place, although the laws varied significantly across states [48]. Despite the proven clinical benefits of naloxone for the reversal of opioid overdoses, its population-level impact depends on the effectiveness of distribution programs alongside multiple contextual factors [49,50]. For this reason, the impact NALs may have had on our results is unclear.

While the goal of this study was to validate the use of help-seeking queries as a surveillance tool across states, our findings call for further investigation within states to contextualize and interpret the results. The inclusion of additional covariates could potentially help to improve the predictive performance of the models developed in this paper and elucidate factors that determine variations in outcomes across years. A key challenge in this regard was the limited sample size, in that there was insufficient statistical power to include additional predictors. One potential remedy to this problem would be to obtain data with more granularity in terms of the time intervals between observations (eg, monthly data) or the geographical level under investigation (eg, county-level data) to increase the number of observations. Finally, while this study retrospectively analyzes SUD help-seeking internet search data to validate their value for surveillance and linkage to treatment, real-time analysis would be the most useful for informing public health agencies, as indicated by some examples investigating mental health–related outcomes during the COVID-19 pandemic [51,52].

### Conclusions

This study examined temporal and spatial trends in the annual fractions of substance use help-seeking internet search queries by US state for cocaine, methamphetamine, opioids, cannabis, and alcohol. Our investigations showed positive, statistically significant associations for the models relating to treatment need (but not receiving treatment) for alcohol use, treatment admissions for opioid and methamphetamine use, and overdose mortality data. In the wake of current substance use trends, it is critical that public health professionals learn from and respond to the millions of individuals searching for help online. The field should invest in and prioritize automated surveillance, including extensions of our approach, to understand evolving public health needs.

## Appendix

Multimedia Appendix 1 Supplementary tables and figures.

## Abbreviations

AHRQ: Agency for Healthcare Quality and Research
API: application programming interface
CRF: case report form
ED: emergency department
NAL: naloxone access law
NSDUH: National Survey on Drug Use and Health
QF: query fraction
RMSE: root mean squared error
SAMHSA: Substance Abuse and Mental Health Services Administration
SUD: substance use disorder
TEDS-A: Treatment Episode Data Set: Admissions
