# Supplementary material for: Characterizing Help-Seeking Searches for Substance Use Treatment From Google Trends and Assessing Their Use for Infoveillance: Longitudinal Descriptive and Validation Statistical Analysis
Source: J Med Internet Res. 2022 Dec 1;24(12):e41527. doi: 10.2196/41527 (PMC9756118; doi:10.2196/41527)

**Multimedia Appendix 1**

Table S1: Results from the analysis of substance-specific QF data (standard errors in brackets)

|  | **Alcohol** | **Cannabis** | **Cocaine** | **Meth** | **Opioids** |
| --- | --- | --- | --- | --- | --- |
| **Intercept** | 10.22  (0.29) | 9.41  (0.10) | 8.18  (0.05) | 8.67  (0.10) | 9.14  (0.05) |
| **Year = 2011** | -0.11  (0.03) | -0.24  (0.05) | -0.28  (0.05) | -0.56  (0.06) | -0.31  (0.05) |
| **Year = 2012** | -0.12  (0.03) | -0.37  (0.05) | -0.43  (0.06) | -0.62  (0.06) | -0.33  (0.05) |
| **Year = 2013** | -0.14  (0.03) | -0.36  (0.05) | -0.53  (0.06) | -0.62  (0.06) | -0.32  (0.05) |
| **Year = 2014** | -0.13  (0.03) | -0.23  (0.05) | -0.54  (0.06) | -0.69  (0.06) | -0.27  (0.05) |
| **Year = 2015** | -0.12  (0.03) | -0.20  (0.05) | -0.44  (0.06) | -0.55  (0.06) | -0.20  (0.05) |
| **Year = 2016** | 0.01  (0.03) | -0.20  (0.05) | -0.47  (0.06) | -0.60  (0.06) | -0.13  (0.05) |
| **Year = 2017** | 0.04  (0.03) | -0.25  (0.05) | -0.42  (0.06) | -0.52  (0.06) | -0.11  (0.05) |
| **Year = 2018** | 0.02  (0.03) | -0.28  (0.05) | -0.40  (0.06) | -0.53  (0.06) | -0.14  (0.05) |
| **Year = 2019** | 0.07  (0.03) | -0.26  (0.05) | -0.50  (0.06) | -0.50  (0.06) | -0.21  (0.05) |
| **Year = 2020** | 0.27  (0.03) | -0.20  (0.05) | -0.32  (0.06) | -0.29  (0.06) | -0.14  (0.05) |

Table S2: Pairwise comparisons from the Bonferroni corrections showing the rate ratio of expected health-seeking search counts for each year relative to the previous year

| **Pairwise Comparison** | **Alcohol** | **Cannabis** | **Cocaine** | **Meth** | **Opioids** |
| --- | --- | --- | --- | --- | --- |
| **2010-2011** | 0.90  (*P*=0.03) | 0.79  (*P*<0.001) | 0.75  (*P*<0.001) | 0.57  (*P*<0.001) | 0.74  (*P*<0.001) |
| **2011-2012** | 0.98  (*P*=1.00) | 0.87  (*P*=0.38) | 0.86  (*P*=0.74) | 0.94  (*P*=1.00) | 0.98  (*P*=1.00) |
| **2012-2013** | 0.99  (*P*=1.00) | 1.01  (*P*=1.00) | 0.91  (*P*=1.00) | 1.00  (*P*=1.00) | 1.01  (*P*=1.00) |
| **2013-2014** | 1.00  (*P*=1.00) | 1.15  (*P*=0.28) | 0.98  (*P*=1.00) | 0.93  (*P*=1.00) | 1.05  (*P*=1.00) |
| **2014-2015** | 1.02  (*P*=1.00) | 1.02  (*P*=1.00) | 1.11  (*P*=1.00) | 1.15  (*P*=1.00) | 1.07  (*P*=1.00) |
| **2015-2016** | 1.13  (*P*=0.003) | 1.00  (*P*=1.00) | 0.97  (*P*=1.00) | 0.95  (*P*=1.00) | 1.07  (*P*=1.00) |
| **2016-2017** | 1.04  (*P*=1.00) | 0.96  (*P*=1.00) | 1.05  (*P*=1.00) | 1.07  (*P*=1.00) | 1.02  (*P*=1.00) |
| **2017-2018** | 0.98  (*P*=1.00) | 0.97  (*P*=1.00) | 1.02  (*P*=1.00) | 1.00  (*P*=1.00) | 0.97  (*P*=1.00) |
| **2018-2019** | 1.05  (*P*=1.00) | 1.02  (*P*=1.00) | 0.91  (*P*=1.00) | 1.03  (*P*=1.00) | 0.93  (*P*=1.00) |
| **2019-2020** | 1.21  (*P*<0.001) | 1.05  (*P*=1.00) | 1.19  (*P*=0.27) | 1.23  (*P*=0.04) | 1.07  (*P*=1.00) |

Table S3: Results from the analysis of the number of people needing but not receiving treatment for illicit drug use (log ratio scale)

|  | **Estimates** | **Standard Errors** | **Pr(>\|z\|)** |
| --- | --- | --- | --- |
| **Intercept** | -3.83 | 0.11 | <0.001 |
| **Combined QF** | 0.01 | 0.01 | 0.26 |
| **Year = 2017** | -0.04 | 0.04 | 0.33 |
| **Year = 2018** | 0.00 | 0.05 | 0.95 |
| **Year = 2019** | 0.07 | 0.05 | 0.14 |

**Table S4**: Results from the analysis of the number of people needing but not receiving treatment for alcohol use

|  | **Estimates** | **Standard Errors** | **Pr(>\|z\|)** |
| --- | --- | --- | --- |
| **Intercept** | -3.09 | 0.05 | <0.001 |
| **Alcohol QF** | 0.01* | 0.00 | 0.003 |
| **Year = 2017** | -0.05 | 0.03 | 0.08 |
| **Year = 2018** | -0.06 | 0.03 | 0.04 |
| **Year = 2019** | -0.08 | 0.03 | 0.004 |

**p-values: <0.05 = ***

**Table S5**: Root Mean Squared Error Estimates for Predicted Rates of People Needing But Not Receiving Treatment for Alcohol Use Years

| **Year** | **Mean RMSE** |
| --- | --- |
| 2016 | 698 |
| 2017 | 689 |
| 2018 | 744 |
| 2019 | 658 |

**Table 6**: Root Mean Squared Error Estimates for Predicted Rates of People Needing But Not Receiving Treatment for Alcohol Use Across States and Territories

| **State** | **Average Rate of People per 100,000** | | | **Average Number of People** | | |
| --- | --- | --- | --- | --- | --- | --- |
|  | **Observed** | **Predicted** | **RMSE** | **Observed** | **Predicted** | **RMSE** |
| **DC** | 8097 | 4925 | 3172 | 49250 | 29939 | 19311 |
| **Colorado** | 6719 | 5082 | 1636 | 329250 | 248620 | 80630 |
| **Oregon** | 6675 | 5053 | 1622 | 242750 | 183700 | 59050 |
| **Montana** | 6704 | 5131 | 1574 | 61500 | 47035 | 14465 |
| **Vermont** | 6373 | 4947 | 1426 | 35500 | 27555 | 7945 |
| **North Dakota** | 6360 | 5010 | 1349 | 41000 | 32304 | 8696 |
| **South Dakota** | 6249 | 4973 | 1276 | 46250 | 36831 | 9419 |
| **Massachusetts** | 6146 | 4989 | 1157 | 372250 | 302270 | 69980 |
| **Rhode Island** | 6032 | 4970 | 1062 | 56500 | 46553 | 9947 |
| **New Hampshire** | 5934 | 4905 | 1029 | 71250 | 58856 | 12394 |
| **Mississippi** | 4053 | 5075 | 1021 | 103750 | 129883 | 26133 |
| **Alaska** | 5986 | 5019 | 968 | 37250 | 31229 | 6021 |
| **West Virginia** | 4066 | 4996 | 930 | 64750 | 79553 | 14803 |
| **Alabama** | 4335 | 5264 | 929 | 183250 | 222408 | 39158 |
| **New Jersey** | 4445 | 5350 | 905 | 345500 | 415804 | 70304 |
| **Wisconsin** | 6193 | 5295 | 898 | 312250 | 266980 | 45270 |
| **Utah** | 4186 | 5044 | 858 | 107250 | 129319 | 22069 |
| **Georgia** | 4083 | 4915 | 832 | 366500 | 440963 | 74463 |
| **North Carolina** | 4260 | 5086 | 825 | 381500 | 454999 | 73499 |
| **Wyoming** | 5728 | 5004 | 724 | 28500 | 24897 | 3603 |
| **Delaware** | 5649 | 4985 | 665 | 47500 | 41898 | 5602 |
| **Connecticut** | 5671 | 5041 | 630 | 178750 | 158898 | 19852 |
| **Iowa** | 6079 | 5457 | 622 | 164500 | 147658 | 16842 |
| **Tennessee** | 4409 | 4962 | 553 | 257250 | 289458 | 32208 |
| **California** | 5669 | 5158 | 550 | 1928000 | 1754016 | 187113 |
| **Texas** | 4369 | 4919 | 549 | 1047250 | 1179728 | 132478 |
| **Florida** | 4434 | 4976 | 543 | 821500 | 923546 | 102046 |
| **Nebraska** | 5487 | 4959 | 528 | 89500 | 80887 | 8613 |
| **Hawaii** | 4872 | 5120 | 494 | 60000 | 63065 | 6083 |
| **Washington** | 5487 | 5005 | 482 | 353500 | 322441 | 31059 |
| **Maine** | 5395 | 4944 | 450 | 64250 | 58882 | 5368 |
| **Arkansas** | 4513 | 4943 | 430 | 116500 | 127562 | 11062 |
| **Louisiana** | 5453 | 5034 | 419 | 217750 | 201025 | 16725 |
| **South Carolina** | 4830 | 5237 | 407 | 212000 | 229797 | 17797 |
| **Illinois** | 5433 | 5031 | 402 | 600000 | 555596 | 44404 |
| **Kentucky** | 4568 | 4903 | 335 | 175750 | 188646 | 12896 |
| **New Mexico** | 5183 | 5005 | 329 | 93250 | 90085 | 5897 |
| **Indiana** | 4812 | 4980 | 289 | 276250 | 285791 | 16608 |
| **Oklahoma** | 5031 | 5258 | 287 | 168750 | 176330 | 9606 |
| **Maryland** | 4827 | 5089 | 274 | 252250 | 265969 | 14339 |
| **Missouri** | 4829 | 5103 | 274 | 255250 | 269731 | 14481 |
| **Nevada** | 5029 | 5076 | 236 | 130750 | 131831 | 6113 |
| **Arizona** | 4777 | 5003 | 226 | 292000 | 305965 | 13965 |
| **Ohio** | 4925 | 4985 | 211 | 497750 | 503981 | 21346 |
| **Minnesota** | 5054 | 4964 | 201 | 242500 | 238143 | 9697 |
| **Pennsylvania** | 4850 | 5017 | 196 | 544000 | 562714 | 21999 |
| **Kansas** | 4936 | 5004 | 180 | 122500 | 124180 | 4444 |
| **New York** | 5105 | 4973 | 177 | 876000 | 853255 | 30463 |
| **Michigan** | 5112 | 5073 | 162 | 444000 | 440637 | 14076 |
| **Virginia** | 5050 | 5129 | 135 | 371250 | 377121 | 9964 |
| **Idaho** | 5015 | 4977 | 133 | 73750 | 73172 | 1937 |

**Red: worst 10%, Green: best 10% in terms of predictive performance**

**Table S7**: Results from the analysis of rates of individual treatment referrals for alcohol use (log rate ratio)

|  | **Estimates** | **Standard Errors** | **Pr(>\|z\|)** |
| --- | --- | --- | --- |
| **Intercept** | -7.40 | 0.17 | <0.001 |
| **Year = 2012** | -0.07 | 0.05 | 0.21 |
| **Year = 2013** | -0.09 | 0.05 | 0.09 |
| **Year = 2014** | -0.22 | 0.05 | <0.001 |
| **Year = 2015** | -0.17 | 0.05 | 0.001 |
| **Year = 2016** | -0.17 | 0.06 | 0.002 |
| **Year = 2017** | -0.06 | 0.06 | 0.29 |
| **Year = 2018** | -0.06 | 0.05 | 0.29 |
| **Alcohol QF** | >0.001 | 0.00 | 0.92 |

**Table S8**: Results from the analysis of rates of individual treatment referrals for cannabis use (log rate ratio)

|  | **Estimates** | **Standard Errors** | **Pr(>\|z\|)** |
| --- | --- | --- | --- |
| **Intercept** | -9.01 | 0.19 | <0.001 |
| **Year = 2012** | -0.06 | 0.06 | 0.38 |
| **Year = 2013** | -0.05 | 0.06 | 0.43 |
| **Year = 2014** | -0.30 | 0.07 | <0.001 |
| **Year = 2015** | -0.25 | 0.07 | <0.001 |
| **Year = 2016** | -0.32 | 0.07 | <0.001 |
| **Year = 2017** | -0.25 | 0.07 | <0.001 |
| **Year = 2018** | -0.39 | 0.07 | <0.001 |
| **Cannabis QF** | 0.03 | 0.02 | 0.07 |

**Table S9**: Results from the analysis of rates of individual treatment referrals for cocaine use (log rate ratio)

|  | **Estimates** | **Standard Errors** | **Pr(>\|z\|)** |
| --- | --- | --- | --- |
| **Intercept** | -8.80 | 0.31 | <0.001 |
| **Year = 2012** | -0.23 | 0.09 | 0.01 |
| **Year = 2013** | -0.32 | 0.10 | 0.001 |
| **Year = 2014** | -0.54 | 0.10 | <0.001 |
| **Year = 2015** | -0.47 | 0.10 | <0.001 |
| **Year = 2016** | -0.48 | 0.10 | <0.001 |
| **Year = 2017** | -0.27 | 0.09 | 0.003 |
| **Year = 2018** | -0.29 | 0.09 | 0.003 |
| **Cocaine QF** | -0.11 | 0.09 | 0.22 |

**Table S10**: Results from the analysis of rates of individual treatment referrals for methamphetamine use (log rate ratio)

|  | **Estimates** | **Standard Errors** | **Pr(>\|z\|)** |
| --- | --- | --- | --- |
| **Intercept** | -10.61 | 0.24 | <0.001 |
| **Year = 2012** | 0.08 | 0.09 | 0.39 |
| **Year = 2013** | 0.43 | 0.08 | <0.001 |
| **Year = 2014** | 0.22 | 0.09 | 0.01 |
| **Year = 2015** | 0.30 | 0.08 | <0.001 |
| **Year = 2016** | 0.47 | 0.08 | <0.001 |
| **Year = 2017** | 0.65 | 0.08 | <0.001 |
| **Year = 2018** | 0.65 | 0.08 | <0.001 |
| **Methamphetamine QF** | 0.23 | 0.04 | <0.001 |

**Table S11**: Results from the analysis of rates of individual treatment referrals for opioid use (log rate ratio)

|  | **Estimates** | **Standard Errors** | **Pr(>\|z\|)** |
| --- | --- | --- | --- |
| **Intercept** | -8.26 | 0.23 | <0.001 |
| **Year = 2012** | 0.01 | 0.08 | 0.91 |
| **Year = 2013** | 0.09 | 0.08 | 0.24 |
| **Year = 2014** | -0.12 | 0.08 | 0.14 |
| **Year = 2015** | 0.09 | 0.08 | 0.30 |
| **Year = 2016** | -0.07 | 0.10 | 0.47 |
| **Year = 2017** | 0.08 | 0.09 | 0.36 |
| **Year = 2018** | 0.08 | 0.09 | 0.36 |
| **Opioid QF** | 0.11 | 0.02 | <0.001 |

**Table S12**: Root Mean Squared Error Estimates for Predicted Rates of Treatment Admissions Stratified by Year

|  | **Methamphetamines** | **Opioids** |
| --- | --- | --- |
| **Year = 2011** | 7.73 | 59.95 |
| **Year = 2012** | 8.59 | 63.55 |
| **Year = 2013** | 11.53 | 68.06 |
| **Year = 2014** | 9.62 | 62.35 |
| **Year = 2015** | 11.28 | 86.59 |
| **Year = 2016** | 13.48 | 83.17 |
| **Year = 2017** | 14.79 | 97.38 |
| **Year = 2018** | 17.07 | 103.80 |

**Table S13a**: Root Mean Squared Error Estimates for Predicted Rates of Treatment Admissions Stratified by State for Methamphetamine Use

|  | **Average Admission Rate per 100,000** | | | **Average Admission Count** | | |
| --- | --- | --- | --- | --- | --- | --- |
| **State** | **Observed** | **Predicted** | **RMSE** | **Observed** | **Predicted** | **RMSE** |
| **Minnesota** | 43.52 | 7.93 | 35.59 | 2431 | 442 | 1989 |
| **Missouri** | 44.62 | 9.45 | 35.17 | 2727 | 578 | 2149 |
| **Arizona** | 43.64 | 9.65 | 33.99 | 3074 | 677 | 2397 |
| **Iowa** | 41.98 | 11.44 | 30.54 | 1321 | 360 | 962 |
| **Oregon** | 36.68 | 6.76 | 29.93 | 1469 | 270 | 1198 |
| **Colorado** | 38.63 | 9.11 | 29.51 | 2178 | 510 | 1667 |
| **California** | 37.43 | 8.07 | 29.35 | 14689 | 3173 | 11515 |
| **West Virginia** | 1.12 | 28.87 | 27.75 | 20 | 526 | 506 |
| **Oklahoma** | 36.01 | 13.56 | 22.44 | 1415 | 533 | 882 |
| **New Mexico** | 3.65 | 22.49 | 18.84 | 77 | 470 | 394 |
| **Arkansas** | 33.75 | 17.99 | 15.76 | 1012 | 539 | 473 |
| **Utah** | 25.79 | 10.38 | 15.41 | 799 | 322 | 477 |
| **Kansas** | 22.23 | 12.96 | 9.27 | 647 | 378 | 270 |
| **Washington** | 13.79 | 8.37 | 7.69 | 1027 | 620 | 580 |
| **Mississippi** | 12.27 | 18.54 | 6.26 | 366 | 553 | 187 |
| **South Carolina** | 2.28 | 8.18 | 5.89 | 115 | 409 | 294 |
| **Maryland** | 0.49 | 5.37 | 4.88 | 30 | 325 | 294 |
| **Pennsylvania** | 0.43 | 5.21 | 4.78 | 55 | 669 | 614 |
| **Kentucky** | 15.89 | 11.85 | 4.78 | 710 | 528 | 214 |
| **Georgia** | 10.95 | 6.85 | 4.10 | 1132 | 709 | 423 |
| **New Jersey** | 0.73 | 4.74 | 4.01 | 66 | 426 | 360 |
| **Alabama** | 5.86 | 9.77 | 3.91 | 288 | 478 | 191 |
| **Wisconsin** | 2.61 | 6.40 | 3.79 | 152 | 372 | 220 |
| **Ohio** | 1.99 | 5.71 | 3.72 | 233 | 667 | 434 |
| **Louisiana** | 11.54 | 8.84 | 3.50 | 538 | 412 | 163 |
| **Tennessee** | 4.81 | 8.23 | 3.42 | 325 | 555 | 230 |
| **Nevada** | 13.84 | 12.48 | 3.08 | 411 | 371 | 92 |
| **Virginia** | 2.35 | 5.13 | 2.78 | 200 | 434 | 234 |
| **Florida** | 2.34 | 5.12 | 2.77 | 492 | 1069 | 577 |
| **Illinois** | 3.69 | 5.17 | 2.47 | 472 | 661 | 316 |
| **North Carolina** | 6.00 | 5.52 | 2.42 | 621 | 566 | 249 |
| **Michigan** | 4.13 | 5.30 | 2.42 | 413 | 529 | 241 |
| **New York** | 2.31 | 4.52 | 2.21 | 458 | 893 | 436 |
| **Texas** | 5.12 | 7.28 | 2.16 | 1442 | 2054 | 612 |
| **Indiana** | 7.64 | 8.67 | 1.93 | 511 | 580 | 129 |

**Red: worst 10%, Green: best 10% in terms of predictive performance**

**Table S13b**: Root Mean Squared Error Estimates for Predicted Rates of Treatment Admissions Stratified by State for Opioid Use

|  | **Average Admission Rate per 100,000** | | | **Average Mortality Count** | | |
| --- | --- | --- | --- | --- | --- | --- |
| **State** | **Observed** | **Predicted** | **RMSE** | **Observed** | **Predicted** | **RMSE** |
| **Maryland** | 592.37 | 61.40 | 530.97 | 35939 | 3709 | 32230 |
| **Massachusetts** | 477.05 | 65.54 | 411.51 | 32618 | 4493 | 28125 |
| **Connecticut** | 381.32 | 65.93 | 315.39 | 13672 | 2364 | 11308 |
| **New York** | 258.93 | 55.34 | 203.60 | 51118 | 10927 | 40191 |
| **Maine** | 282.82 | 86.79 | 196.03 | 3778 | 1161 | 2617 |
| **Delaware** | 312.19 | 137.12 | 175.07 | 3008 | 1311 | 1696 |
| **New Jersey** | 199.60 | 60.77 | 138.84 | 17938 | 5458 | 12480 |
| **Michigan** | 143.20 | 54.75 | 88.45 | 14288 | 5455 | 8833 |
| **Idaho** | 5.39 | 90.97 | 85.58 | 91 | 1546 | 1455 |
| **New Mexico** | 17.34 | 89.87 | 72.53 | 363 | 1880 | 1517 |
| **Oregon** | 113.84 | 51.11 | 62.74 | 4558 | 2042 | 2515 |
| **South Carolina** | 4.67 | 56.75 | 52.08 | 226 | 2833 | 2607 |
| **West Virginia** | 67.02 | 118.42 | 51.40 | 1221 | 2160 | 939 |
| **Wisconsin** | 7.52 | 57.47 | 49.95 | 434 | 3334 | 2899 |
| **Washington** | 14.44 | 60.16 | 47.81 | 1043 | 4434 | 3543 |
| **Kansas** | 11.95 | 58.39 | 46.43 | 348 | 1700 | 1352 |
| **Mississippi** | 19.37 | 65.80 | 46.43 | 577 | 1963 | 1385 |
| **Minnesota** | 90.97 | 48.49 | 42.48 | 5056 | 2696 | 2359 |
| **California** | 86.96 | 45.48 | 41.48 | 34108 | 17842 | 16265 |
| **Nevada** | 20.26 | 61.03 | 40.77 | 600 | 1808 | 1208 |
| **Pennsylvania** | 26.04 | 66.63 | 40.59 | 3336 | 8547 | 5210 |
| **Texas** | 9.35 | 47.14 | 37.79 | 2579 | 13201 | 10622 |
| **District of Columbia** | 102.29 | 70.10 | 36.68 | 703 | 481 | 253 |
| **New Hampshire** | 61.50 | 97.32 | 35.82 | 826 | 1310 | 483 |
| **Arizona** | 83.40 | 67.70 | 35.01 | 5880 | 4735 | 2485 |
| **Alabama** | 27.71 | 57.14 | 33.77 | 1367 | 2795 | 1647 |
| **Arkansas** | 34.82 | 68.04 | 33.22 | 1041 | 2037 | 996 |
| **Georgia** | 20.30 | 51.41 | 31.11 | 2091 | 5297 | 3206 |
| **Colorado** | 82.35 | 51.35 | 31.00 | 4614 | 2864 | 1750 |
| **Oklahoma** | 31.76 | 59.40 | 27.64 | 1246 | 2328 | 1082 |
| **Indiana** | 37.29 | 61.09 | 23.80 | 2484 | 4076 | 1592 |
| **Iowa** | 30.25 | 52.04 | 21.79 | 950 | 1632 | 682 |
| **Kentucky** | 88.48 | 68.28 | 20.19 | 3934 | 3038 | 895 |
| **Tennessee** | 70.80 | 62.25 | 20.09 | 4748 | 4172 | 1334 |
| **Louisiana** | 42.36 | 59.74 | 20.03 | 1973 | 2784 | 934 |
| **Virginia** | 34.00 | 49.59 | 17.65 | 2871 | 4194 | 1498 |
| **North Carolina** | 72.04 | 55.96 | 17.18 | 7393 | 5724 | 1781 |
| **Florida** | 56.87 | 50.52 | 16.13 | 11746 | 10487 | 3315 |
| **Utah** | 67.05 | 60.05 | 15.05 | 2079 | 1853 | 466 |
| **Missouri** | 71.89 | 59.70 | 12.43 | 4390 | 3646 | 759 |
| **Ohio** | 70.77 | 65.59 | 11.23 | 8246 | 7650 | 1307 |
| **Illinois** | 47.26 | 50.90 | 10.38 | 6054 | 6517 | 1330 |

**Red: worst 10%, Green: best 10% in terms of predictive performance**

**Table S14**: Estimates of the rate ratio change in the rates of emergency department visits related to opioid use associated with a one unit increase in the opioid QF variable

|  | **Estimates** | **Lower 95% CI** | **Upper 95% CI** |
| --- | --- | --- | --- |
| **Year = 2011** | 1.06 | 1.03 | 1.10 |
| **Year = 2012** | 1.04 | 1.01 | 1.07 |
| **Year = 2013** | 1.04 | 1.00 | 1.08 |
| **Year = 2014** | 1.03 | 1.00 | 1.07 |
| **Year = 2015** | 1.04 | 1.01 | 1.07 |
| **Year = 2016** | 1.02 | 1.00 | 1.04 |
| **Year = 2017** | 1.01 | 0.98 | 1.03 |
| **Year = 2018** | 1.00 | 0.98 | 1.02 |

**Table S15**: Results from the analysis of rates of emergency department visits related to opioid use (log rate ratio)

|  | **Estimates** | **Standard Errors** | **Pr(>\|z\|)** |
| --- | --- | --- | --- |
| **Intercept** | 4.40 | 0.14 | <0.001 |
| **Year = 2012** | 0.25 | 0.14 | 0.06 |
| **Year = 2013** | 0.31 | 0.15 | 0.04 |
| **Year = 2014** | 0.45 | 0.15 | 0.002 |
| **Year = 2015** | 0.56 | 0.13 | <0.001 |
| **Year = 2016** | 0.94 | 0.13 | <0.001 |
| **Year = 2017** | 1.04 | 0.14 | <0.001 |
| **Year = 2018** | 1.02 | 0.13 | <0.001 |
| **Opioid QF** | 0.06 | 0.02 | <0.001 |
| **(Opioid QF) x (Year = 2012)** | -0.02 | 0.02 | 0.27 |
| **(Opioid QF) x (Year = 2013)** | -0.02 | 0.02 | 0.29 |
| **(Opioid QF) x (Year = 2014)** | -0.03 | 0.02 | 0.16 |
| **(Opioid QF) x (Year = 2015)** | -0.02 | 0.02 | 0.25 |
| **(Opioid QF) x (Year = 2016)** | -0.04 | 0.02 | 0.02 |
| **(Opioid QF) x (Year = 2017)** | -0.06 | 0.02 | 0.003 |
| **(Opioid QF) x (Year = 2018)** | -0.06 | 0.02 | <0.001 |

**Table S16**: Results from the analysis of number of overdose deaths (log rate ratio)

|  | **Estimates** | **Standard Errors** | **Pr(>\|z\|)** |
| --- | --- | --- | --- |
| **Intercept** | -10.19 | 0.10 | <0.001 |
| **Year = 2012** | 0.02 | 0.05 | 0.73 |
| **Year = 2013** | 0.06 | 0.05 | 0.20 |
| **Year = 2014** | 0.15 | 0.05 | 0.001 |
| **Year = 2015** | 0.24 | 0.05 | <0.001 |
| **Year = 2016** | 0.38 | 0.05 | <0.001 |
| **Year = 2017** | 0.48 | 0.05 | <0.001 |
| **Year = 2018** | 0.50 | 0.05 | <0.001 |
| **Opioid QF** | 0.11 | 0.01 | <0.001 |

**Table S17**: Root Mean Squared Error Estimates for Predicted Rates of Overdose Mortality Rates Stratified by Year

|  | **RMSE** |
| --- | --- |
| **Year = 2011** | 3.31 |
| **Year = 2012** | 3.15 |
| **Year = 2013** | 3.01 |
| **Year = 2014** | 3.39 |
| **Year = 2015** | 4.12 |
| **Year = 2016** | 5.28 |
| **Year = 2017** | 6.33 |
| **Year = 2018** | 6.00 |

**Table S18**: Root Mean Squared Error Estimates for Predicted Overdose Mortality Rates Stratified by State

|  | **Average Mortality Rate per 100,000** | | | **Average Mortality Count** | | |
| --- | --- | --- | --- | --- | --- | --- |
| **State** | **Observed** | **Predicted** | **RMSE** | **Observed** | **Predicted** | **RMSE** |
| **West Virginia** | 33.74 | 19.88 | 13.86 | 620 | 365 | 254 |
| **Ohio** | 21.66 | 11.63 | 10.04 | 2518 | 1351 | 1167 |
| **Idaho** | 5.44 | 15.02 | 9.58 | 91 | 249 | 158 |
| **Maryland** | 20.52 | 10.95 | 9.57 | 1231 | 656 | 575 |
| **Massachusetts** | 19.93 | 11.58 | 8.34 | 1354 | 786 | 568 |
| **Kentucky** | 19.10 | 12.01 | 7.10 | 845 | 532 | 313 |
| **Mississippi** | 4.57 | 11.65 | 7.08 | 137 | 348 | 211 |
| **District of Columbia** | 17.75 | 12.68 | 6.51 | 120 | 86 | 44 |
| **Delaware** | 17.46 | 22.66 | 6.37 | 165 | 213 | 59 |
| **New Hampshire** | 22.39 | 16.84 | 5.93 | 299 | 225 | 79 |
| **Arkansas** | 6.04 | 11.83 | 5.79 | 180 | 353 | 173 |
| **Connecticut** | 16.80 | 11.67 | 5.72 | 602 | 418 | 205 |
| **Kansas** | 5.24 | 10.28 | 5.04 | 152 | 298 | 147 |
| **Alabama** | 5.67 | 10.02 | 4.35 | 276 | 487 | 211 |
| **Utah** | 14.68 | 10.45 | 4.31 | 437 | 314 | 126 |
| **Louisiana** | 6.17 | 10.46 | 4.28 | 287 | 486 | 199 |
| **Nevada** | 14.35 | 10.60 | 4.02 | 411 | 306 | 112 |
| **Oklahoma** | 11.60 | 10.29 | 4.02 | 449 | 400 | 156 |
| **Tennessee** | 14.72 | 10.86 | 3.85 | 973 | 718 | 254 |
| **New Jersey** | 12.99 | 10.66 | 3.77 | 1161 | 952 | 337 |
| **Texas** | 4.60 | 8.26 | 3.66 | 1254 | 2262 | 1008 |
| **New Mexico** | 15.92 | 15.25 | 3.58 | 332 | 318 | 75 |
| **Michigan** | 13.18 | 9.63 | 3.55 | 1309 | 956 | 353 |
| **Iowa** | 5.52 | 9.02 | 3.50 | 172 | 281 | 109 |
| **Maine** | 15.61 | 14.90 | 3.18 | 208 | 199 | 42 |
| **California** | 5.24 | 8.06 | 2.82 | 2033 | 3137 | 1104 |
| **Washington** | 9.77 | 10.58 | 2.76 | 698 | 763 | 200 |
| **Illinois** | 11.65 | 8.96 | 2.69 | 1494 | 1150 | 344 |
| **Oregon** | 8.48 | 9.71 | 2.66 | 340 | 393 | 109 |
| **Minnesota** | 6.15 | 8.60 | 2.45 | 337 | 473 | 135 |
| **North Carolina** | 12.24 | 9.95 | 2.29 | 1234 | 1003 | 231 |
| **Pennsylvania** | 12.29 | 11.79 | 1.97 | 1572 | 1508 | 252 |
| **Georgia** | 7.30 | 9.26 | 1.96 | 745 | 947 | 201 |
| **Missouri** | 12.52 | 10.57 | 1.95 | 761 | 643 | 118 |
| **Indiana** | 9.72 | 10.81 | 1.78 | 644 | 716 | 118 |
| **Colorado** | 9.10 | 9.11 | 1.78 | 493 | 497 | 97 |
| **Arizona** | 10.37 | 11.88 | 1.78 | 710 | 815 | 123 |
| **South Carolina** | 10.16 | 10.01 | 1.73 | 500 | 491 | 84 |
| **Wisconsin** | 11.77 | 10.14 | 1.70 | 679 | 585 | 98 |
| **Virginia** | 10.30 | 8.84 | 1.46 | 862 | 740 | 122 |
| **Florida** | 10.22 | 8.93 | 1.46 | 2081 | 1814 | 299 |
| **New York** | 11.24 | 9.84 | 1.40 | 2213 | 1938 | 275 |

**Red: worst 10%, Green: best 10% in terms of predictive performance**


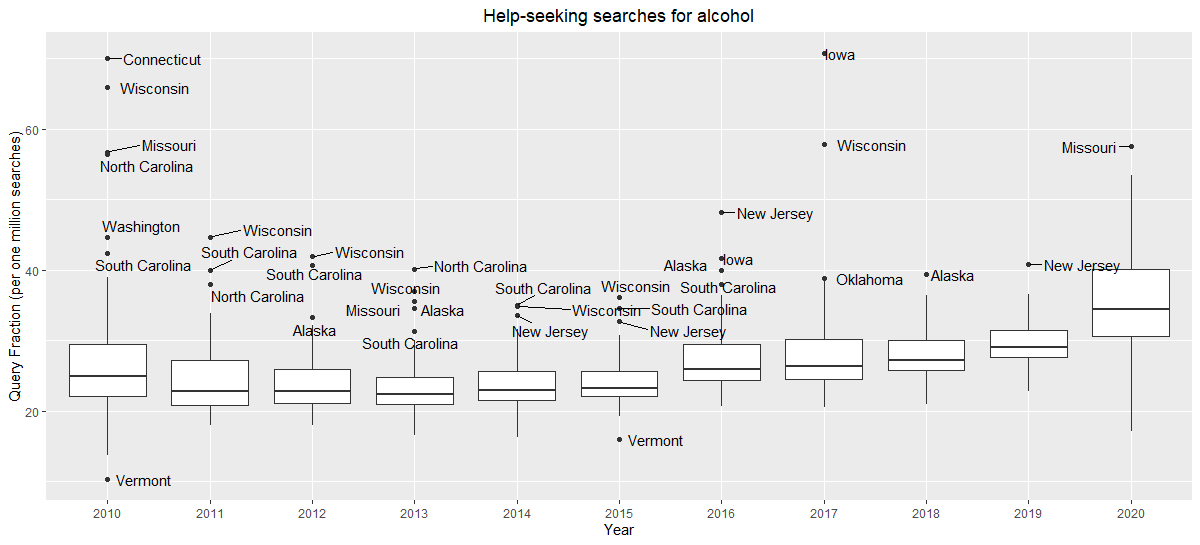
**Figure S1A**: Box and whisker plot of help-seeking searches for alcohol use


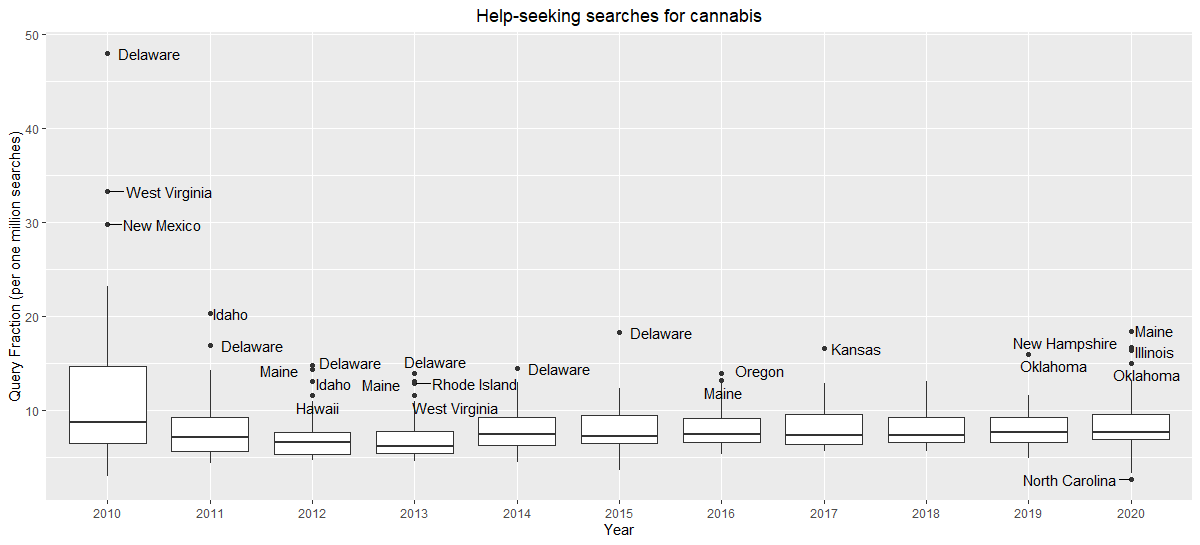
**Figure S1B**: Box and whisker plot of help-seeking searches for cannabis use


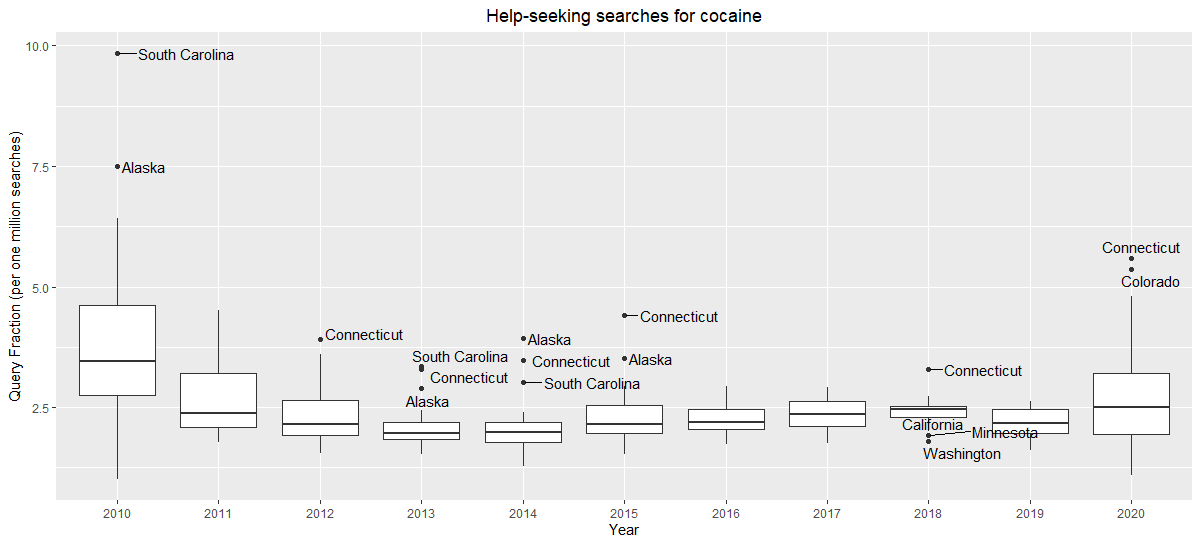
**Figure S1C**: Box and whisker plot of help-seeking searches for cocaine use

**Figure S1D**: Box and whisker plot of help-seeking searches for methamphetamine use


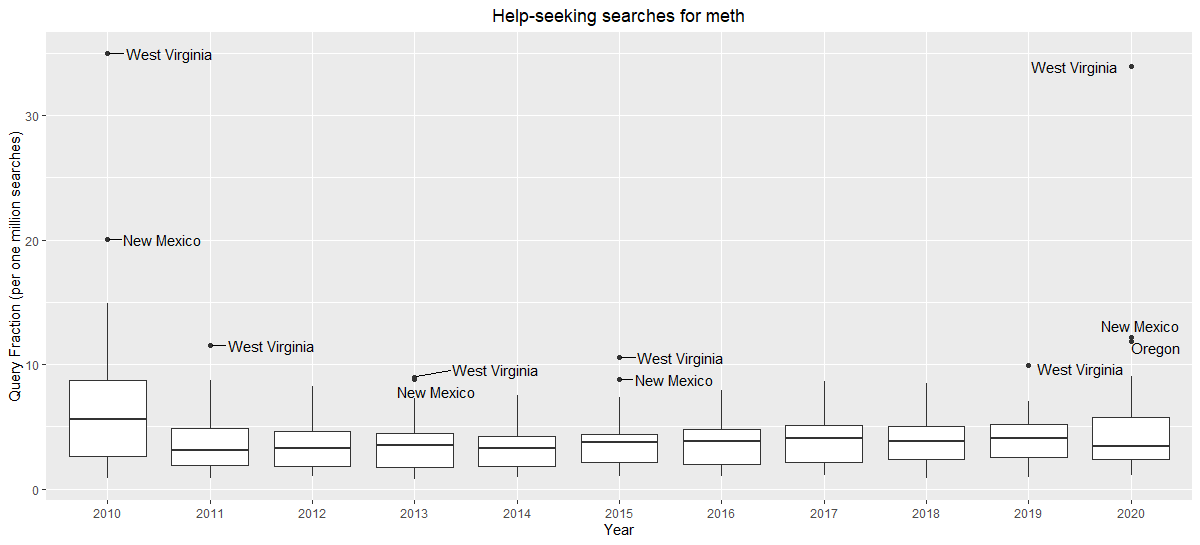


**Figure S2A**: People needing but not receiving treatment for alcohol use


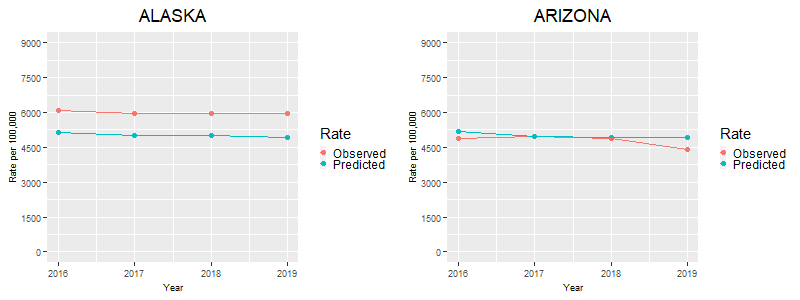

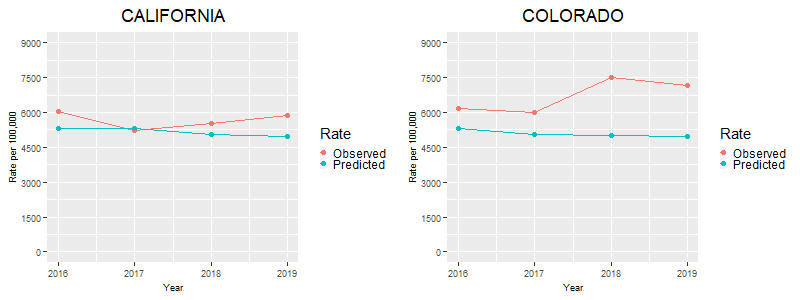

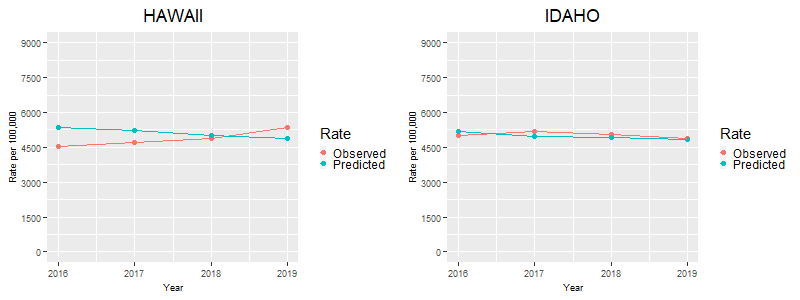

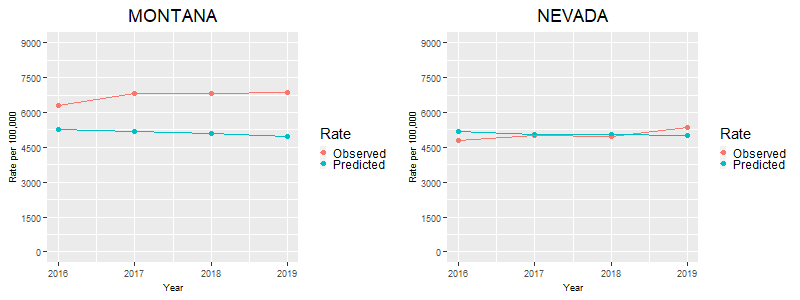

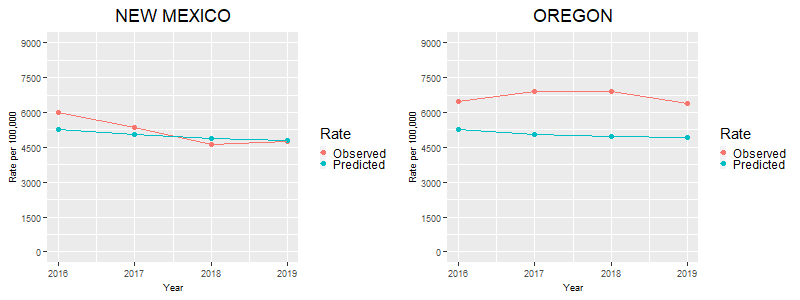

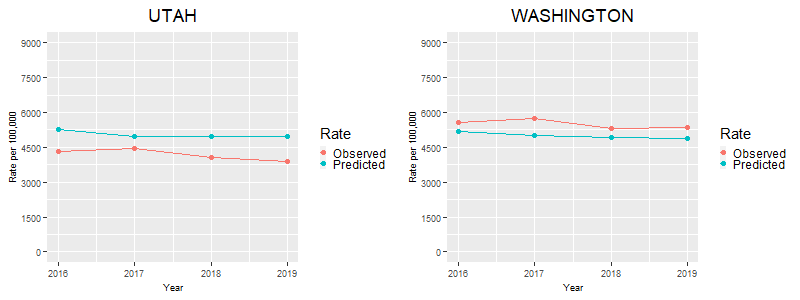


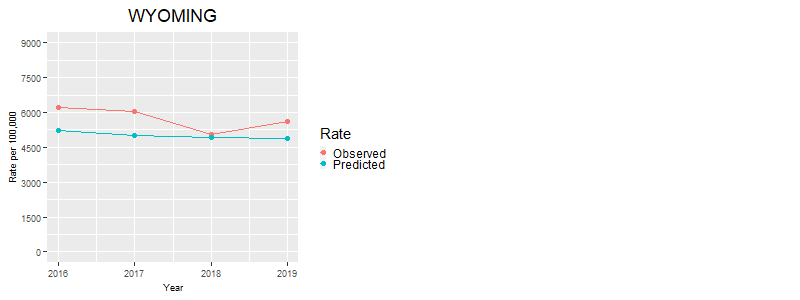


**Figure S2B**: People needing but not receiving treatment for alcohol use (Southern States)


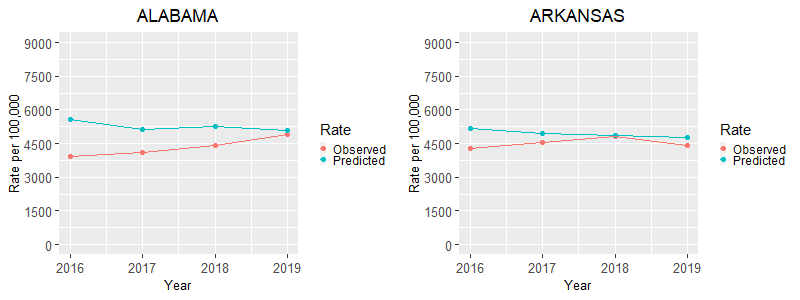

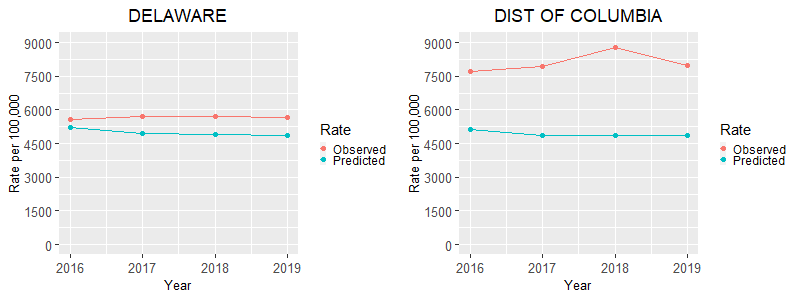

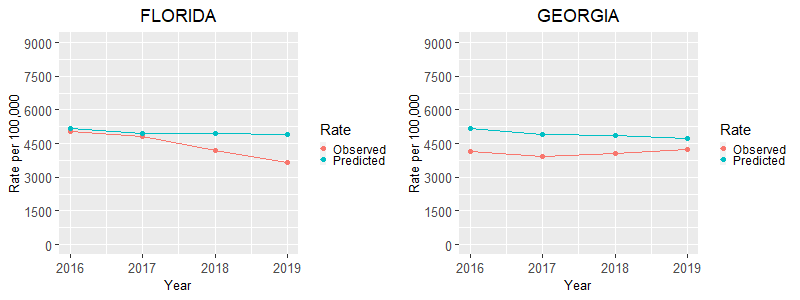

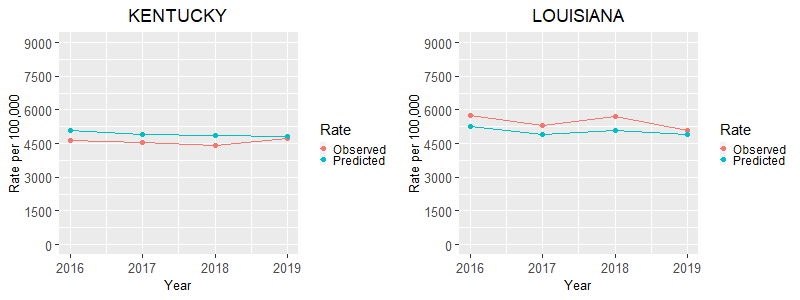

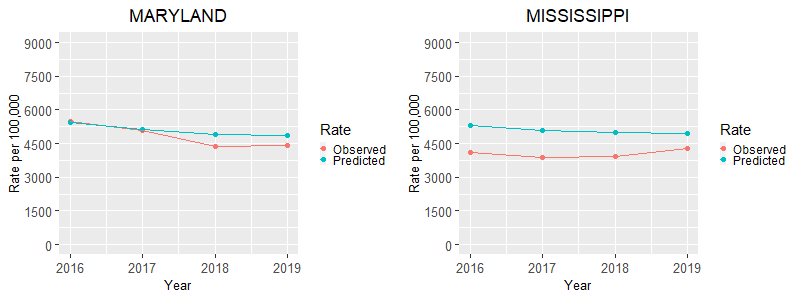

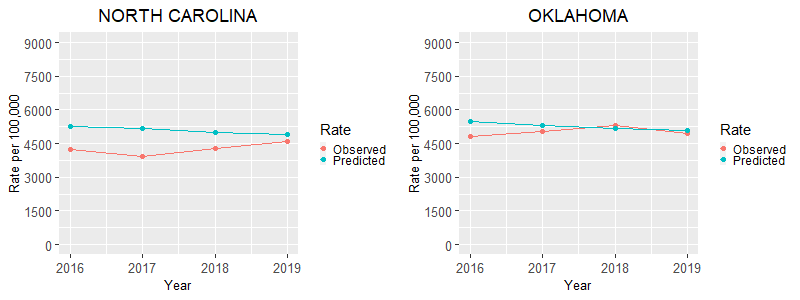

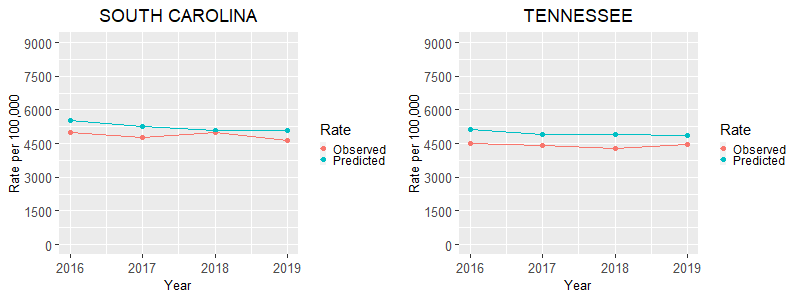

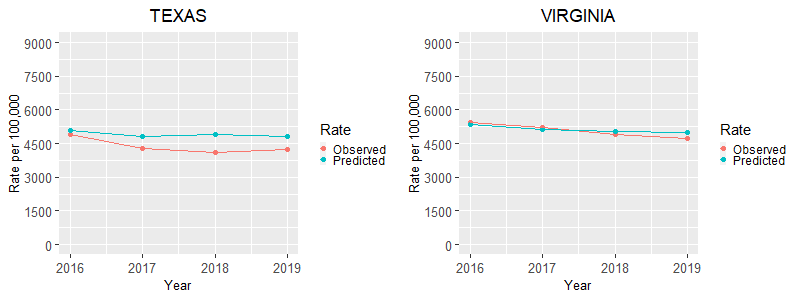

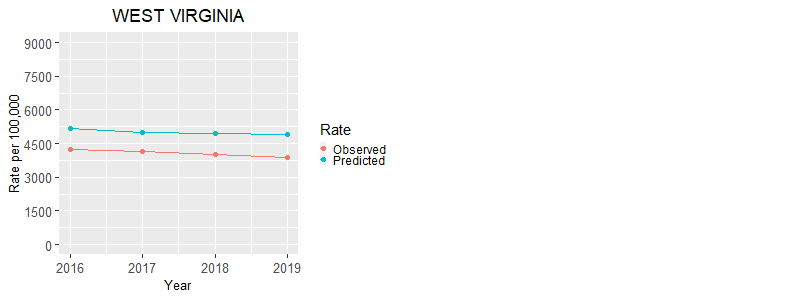


**Figure S2C: People needing but not receiving treatment for alcohol use (Midwestern States)**


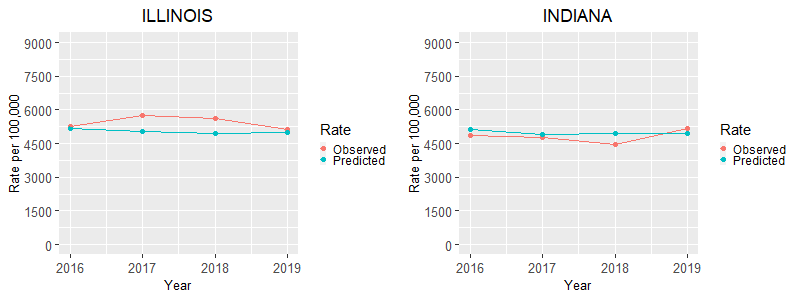

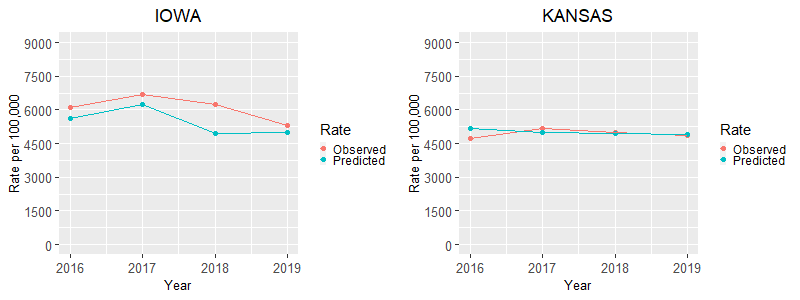

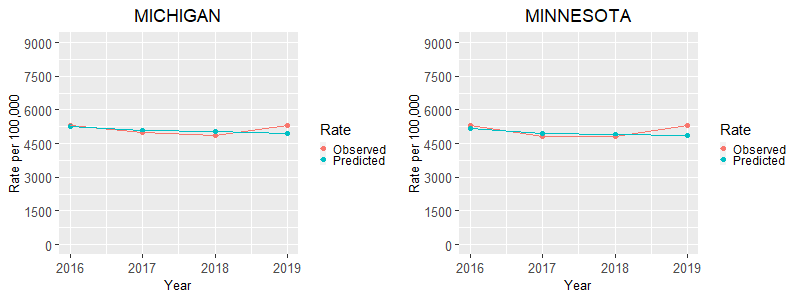

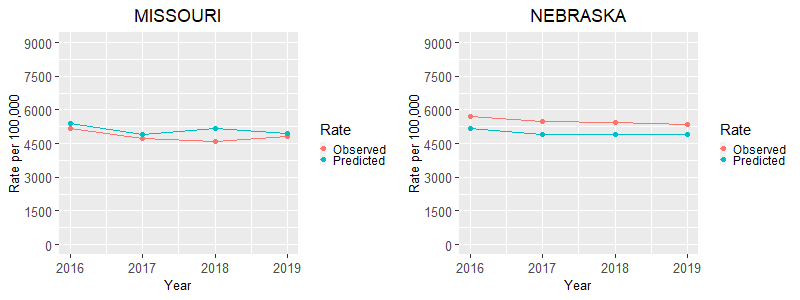

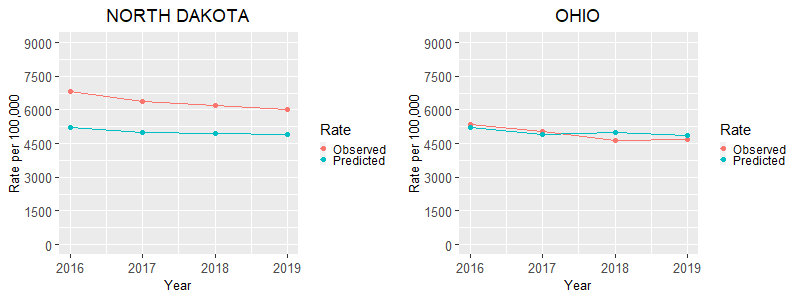

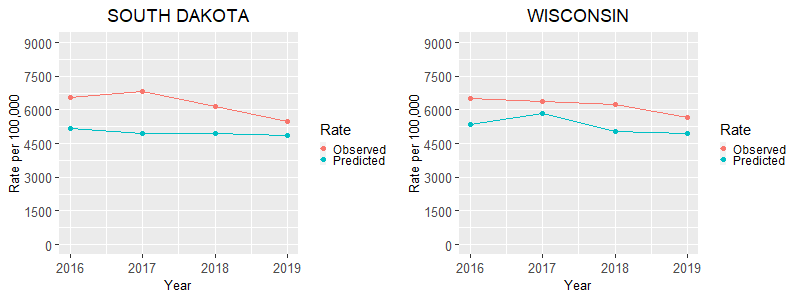


**Figure S2D: People needing but not receiving treatment for alcohol use (Northeastern States)**


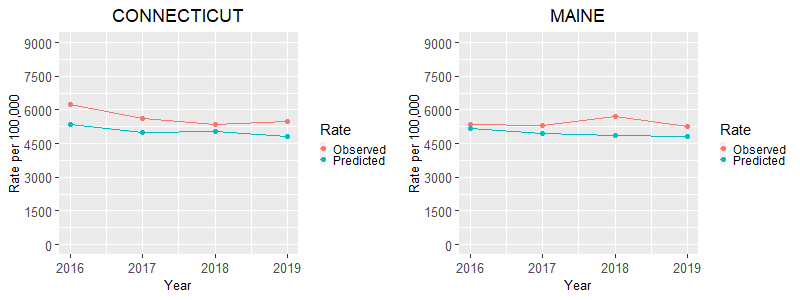

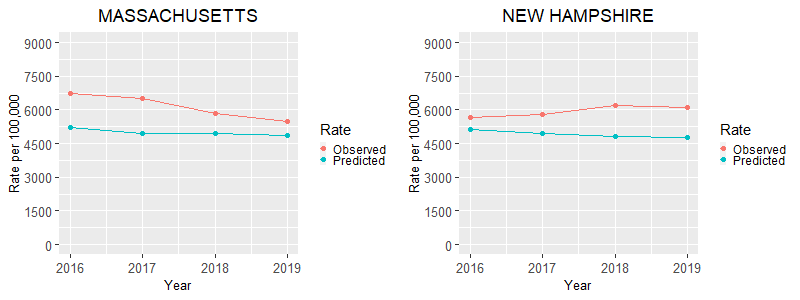

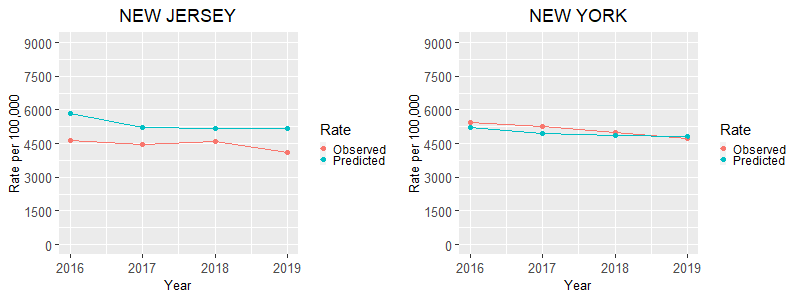

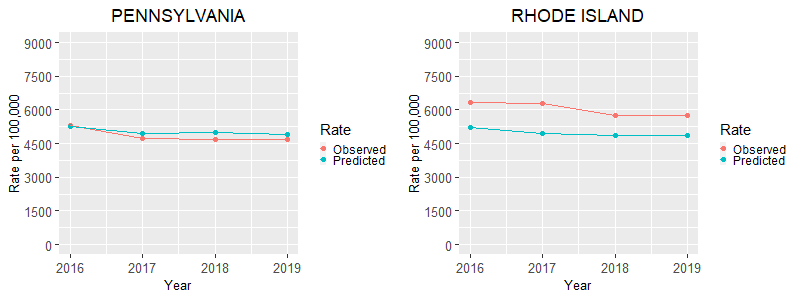

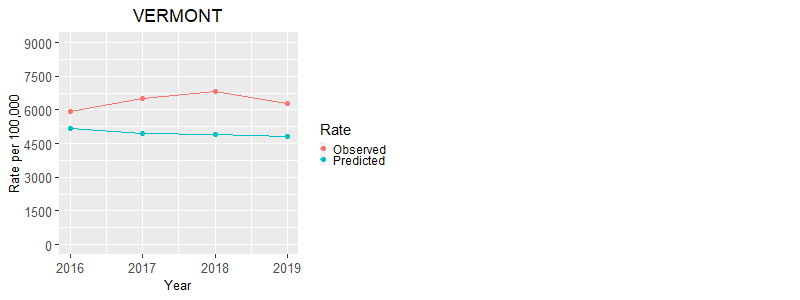


**Figure S3A**: Individual Referrals for Treatment for Methamphetamine Use (Midwestern States)


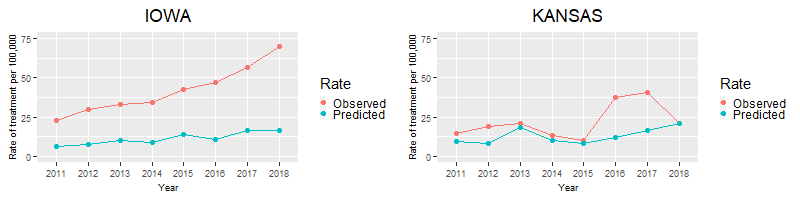

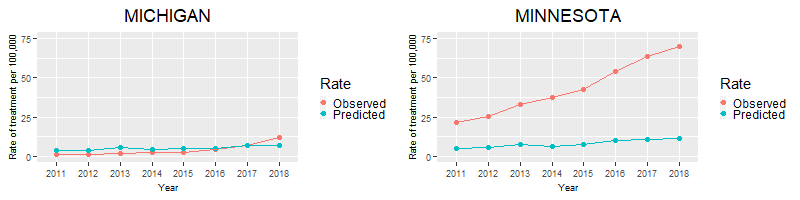

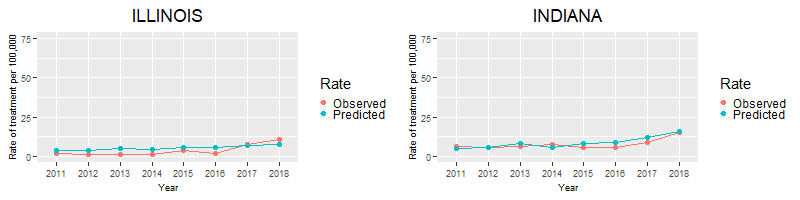

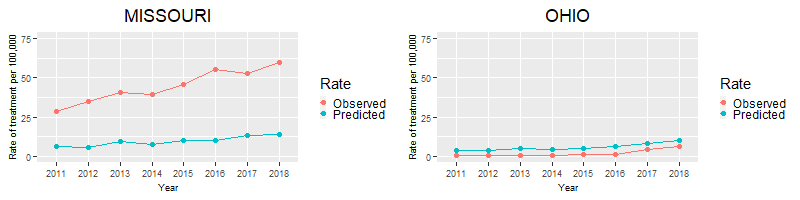

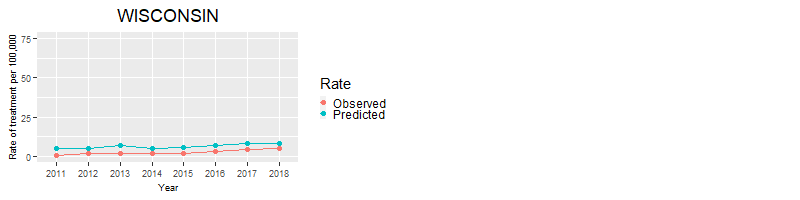


**Figure S3B**: Individual Referrals for Treatment for Methamphetamine Use (Northeastern States)


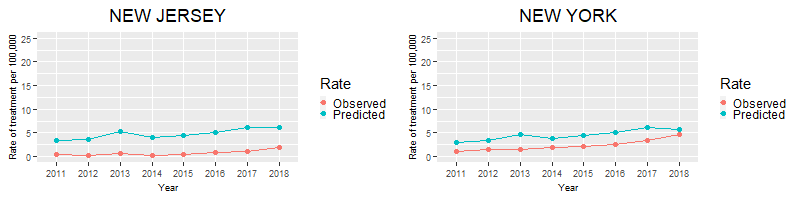

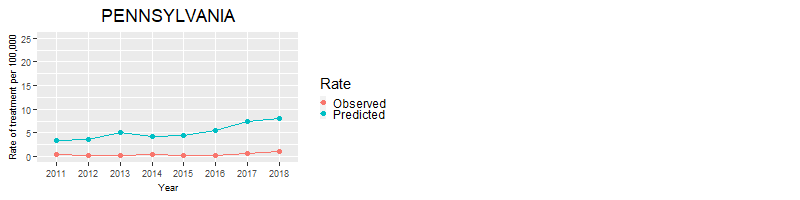


**Figure S3C**: Individual Referrals for Treatment for Methamphetamine Use (Southern States)


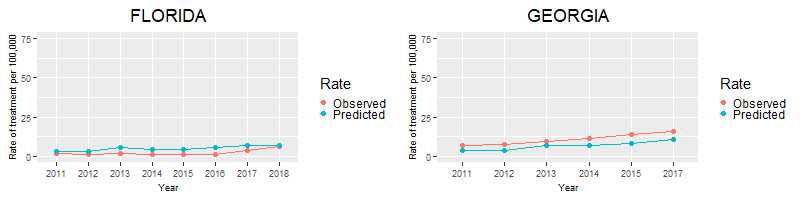

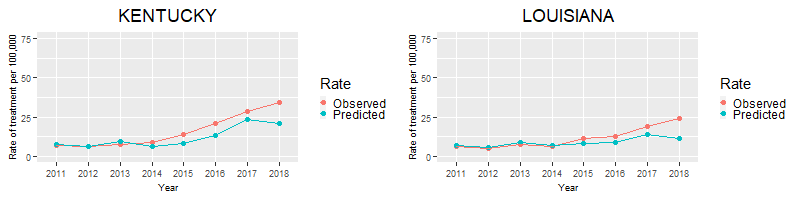

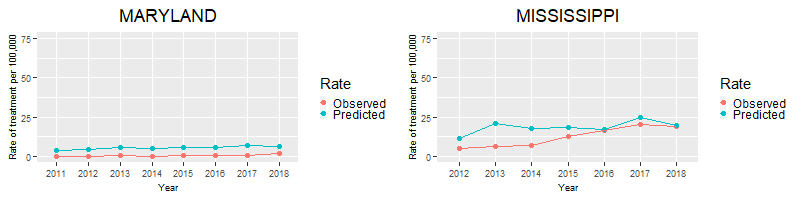

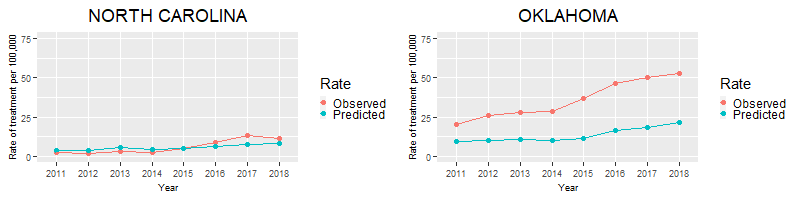

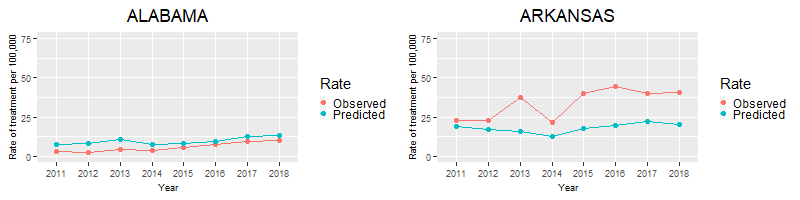

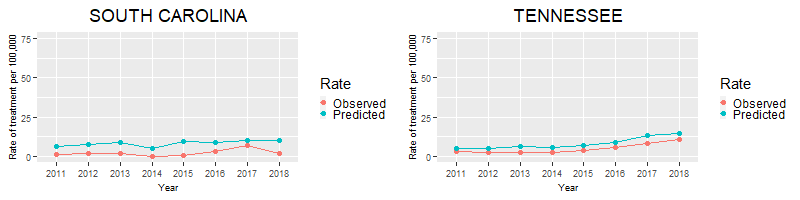

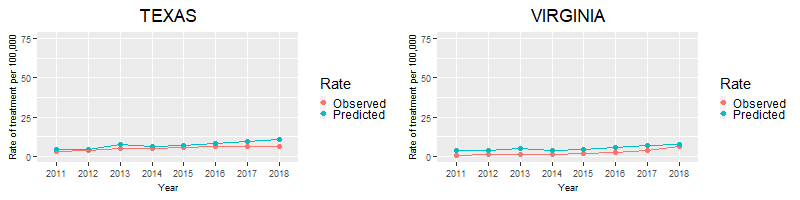

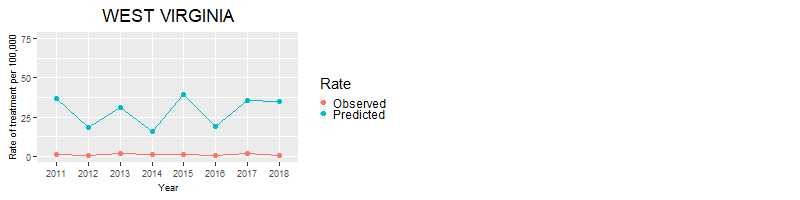


**Figure S3D**: Individual Referrals for Treatment for Methamphetamine Use (Western States)


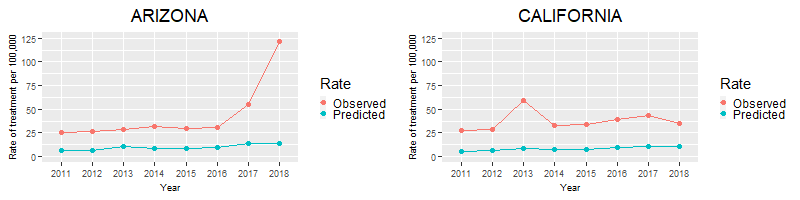

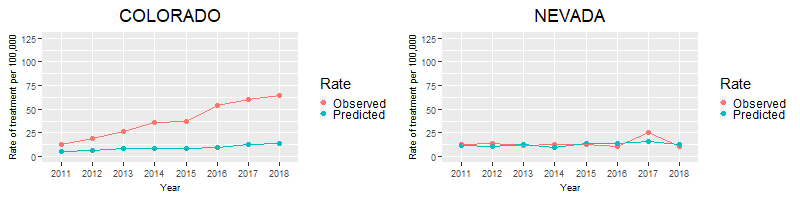

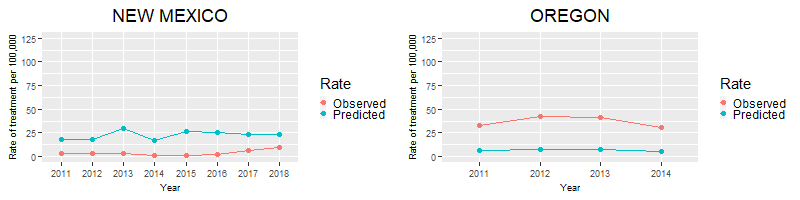

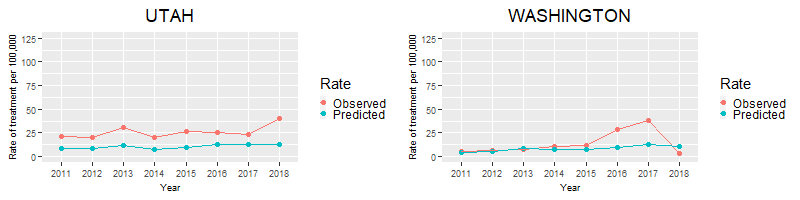


**Figure S4A:** Individual Referrals for Treatment for Opioid Use (Midwestern States)


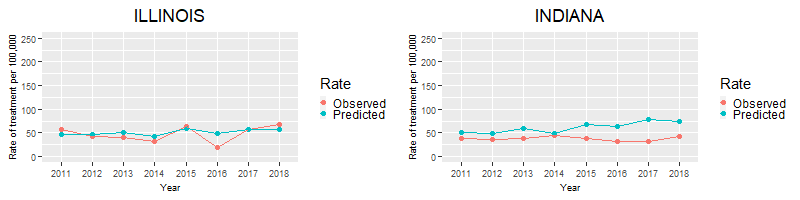

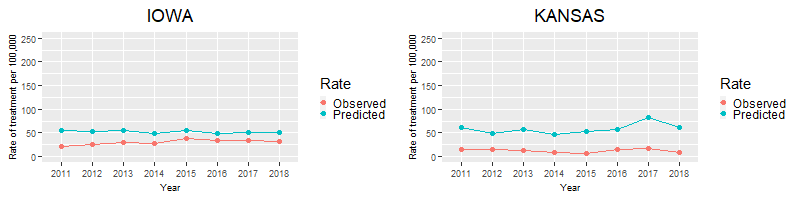

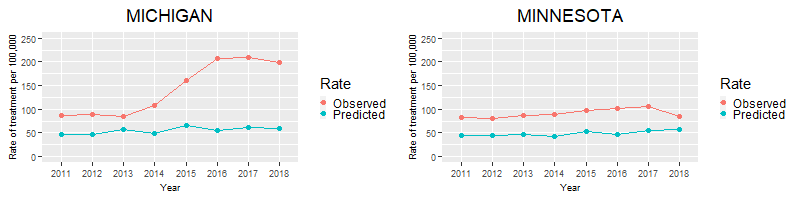

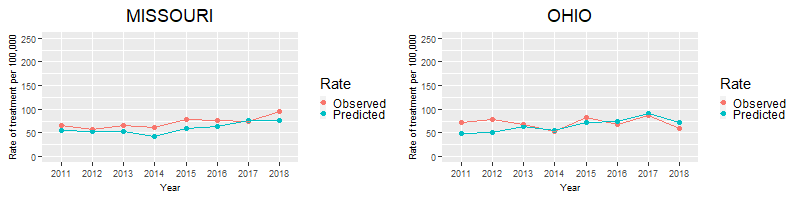

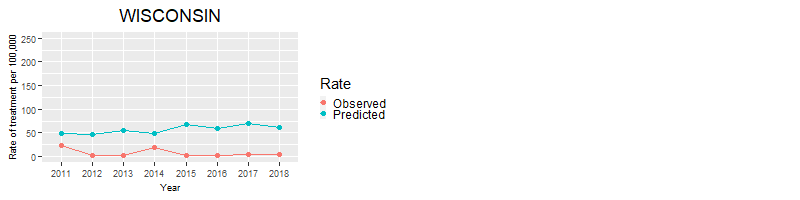


**Figure S4B:** Individual Referrals for Treatment for Opioid Use (Northeastern States)


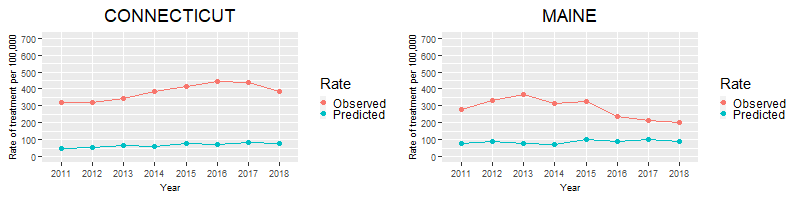

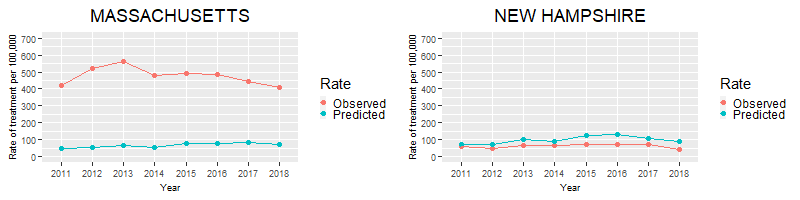

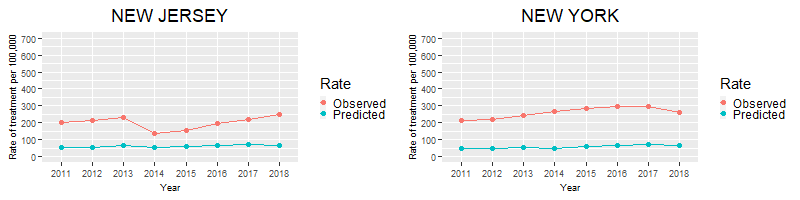

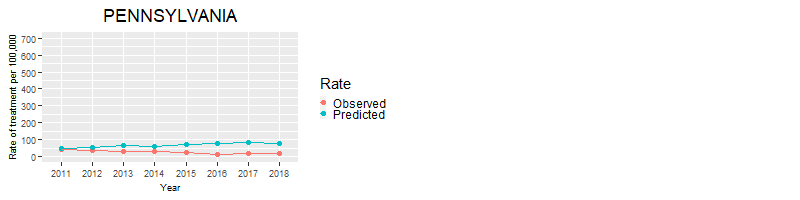


**Figure S4C**: Individual Referrals for Treatment for Opioid Use (Southern States)


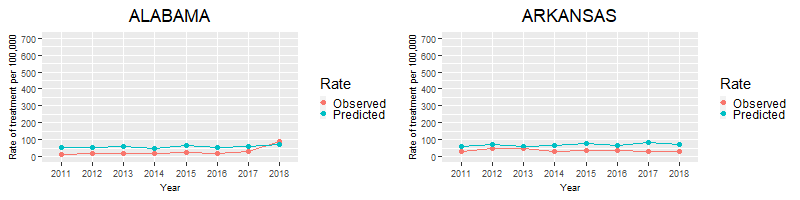

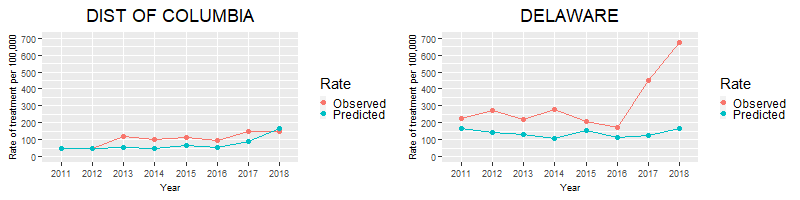

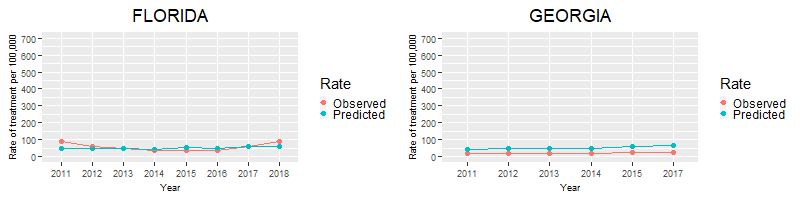

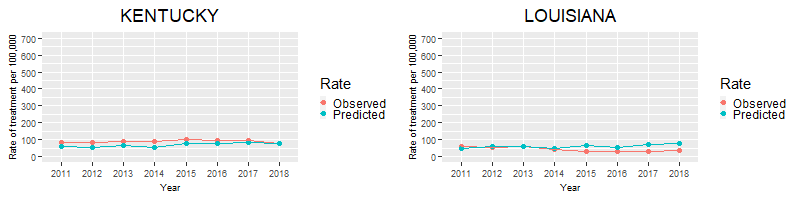

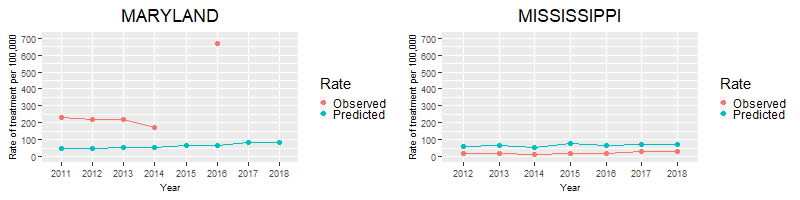

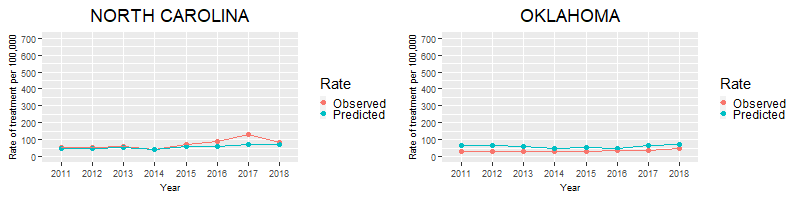

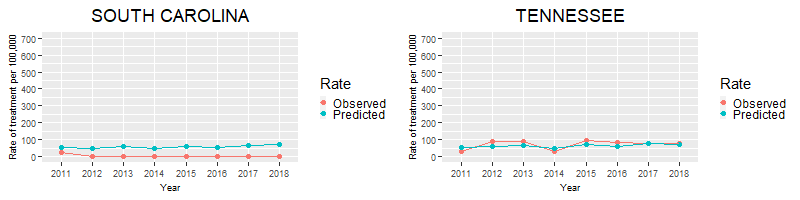

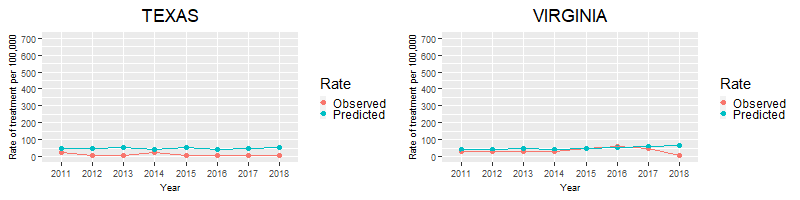

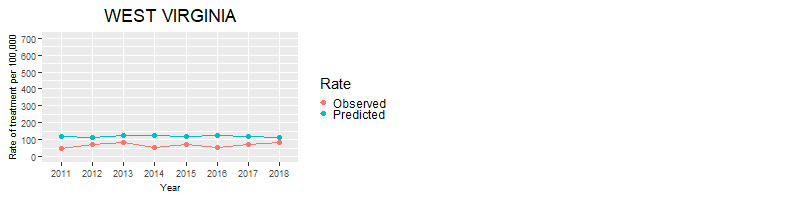


**Figure S4D**: Individual Referrals for Treatment for Opioid Use (Western States)


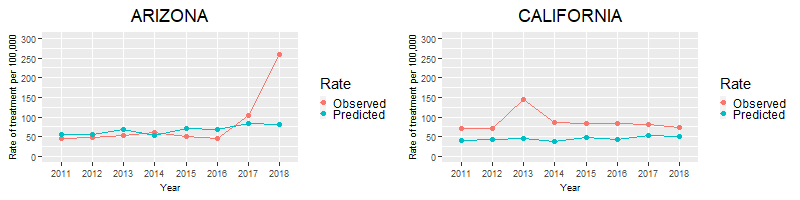

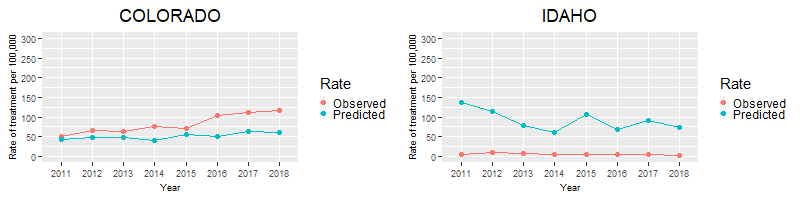

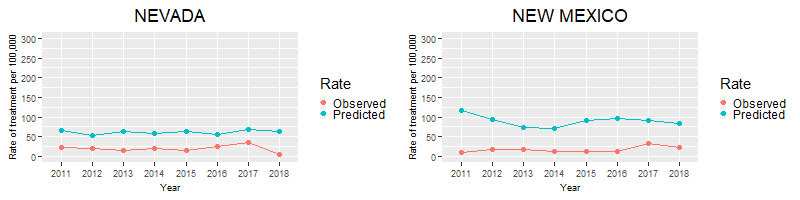

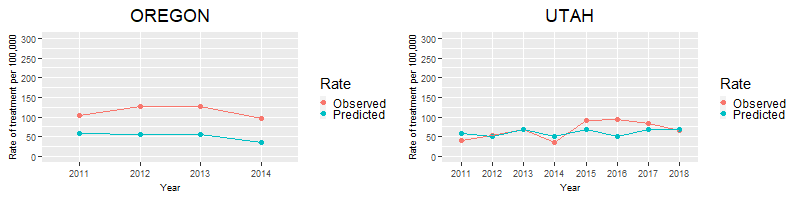

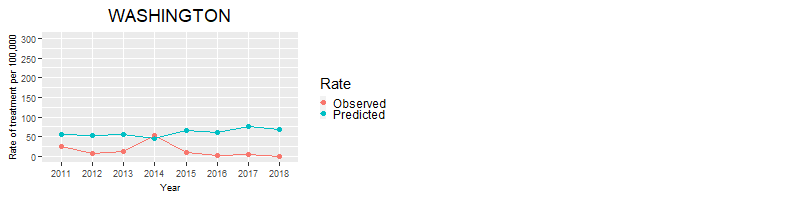


**Figure S5A**: Overdose mortality rates (Midwestern States)


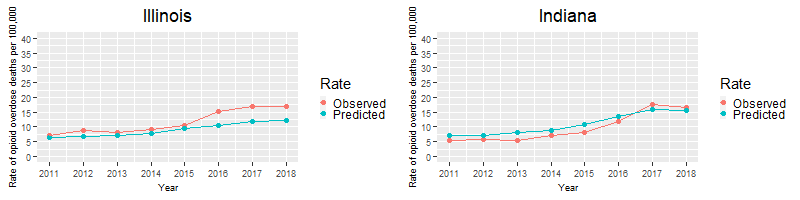

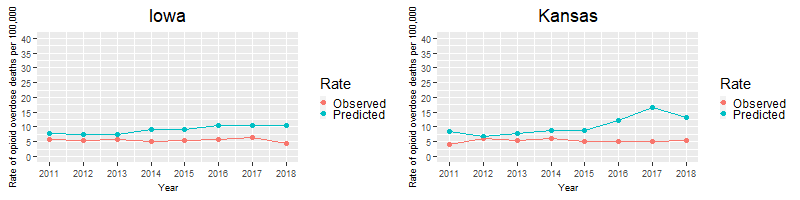

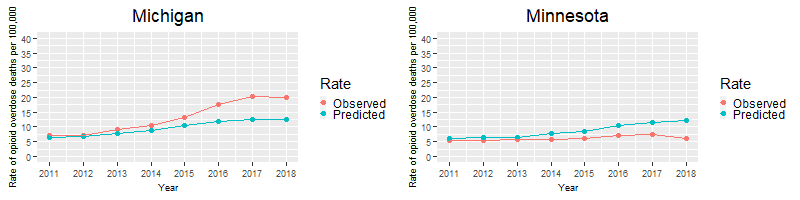

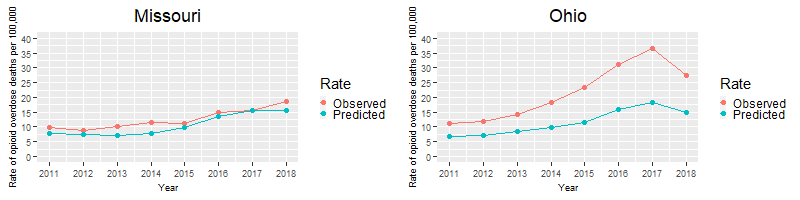

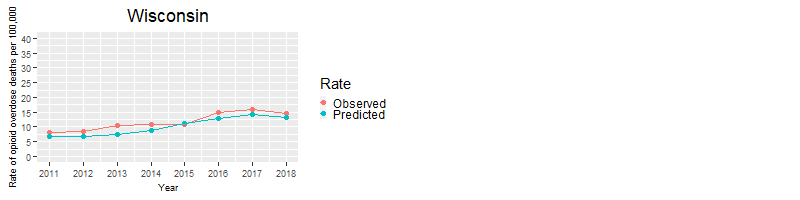


**Figure S5B**: Overdose mortality rates (Northeastern States)


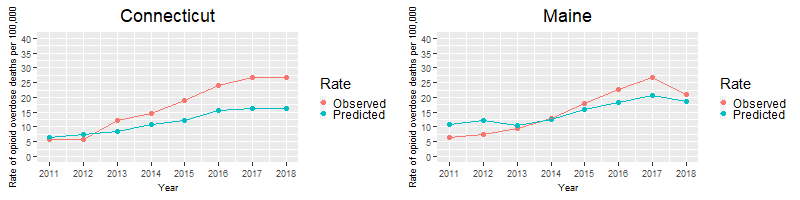

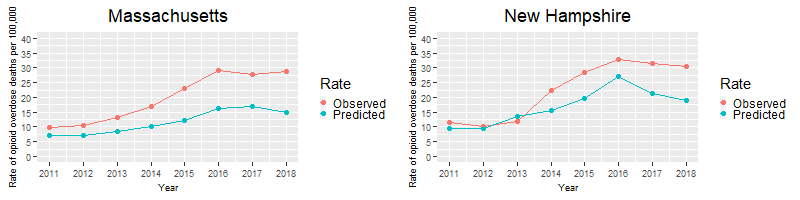

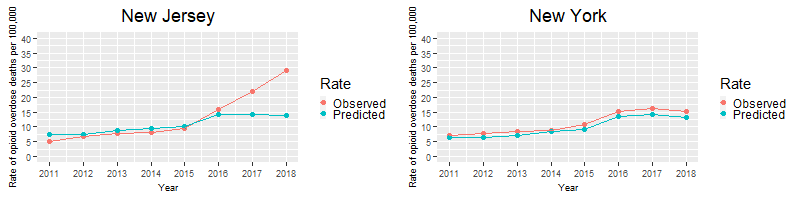

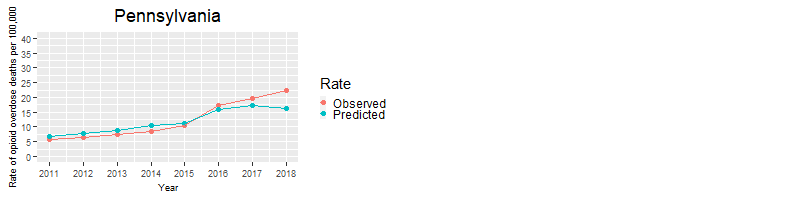


**Figure S5C**: Overdose mortality rates (Southern States)


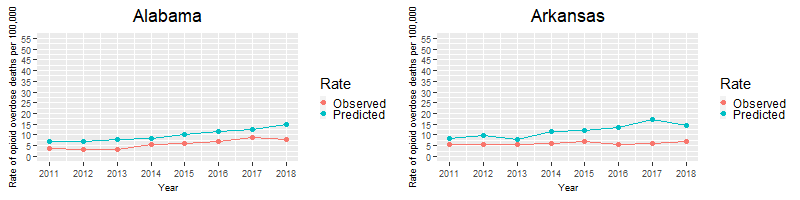

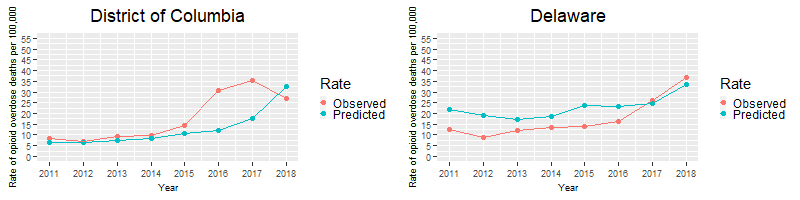

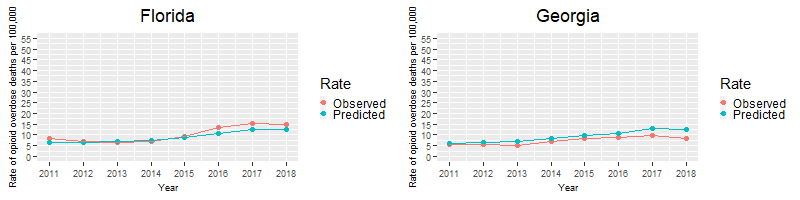

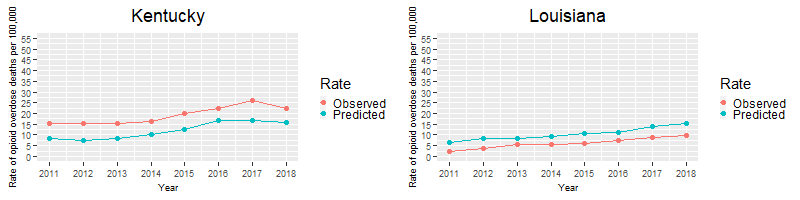

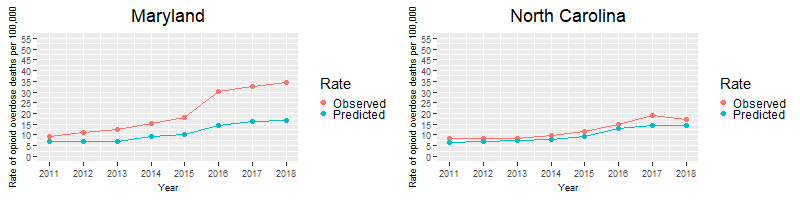

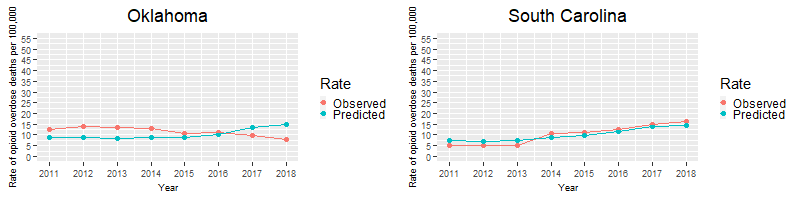

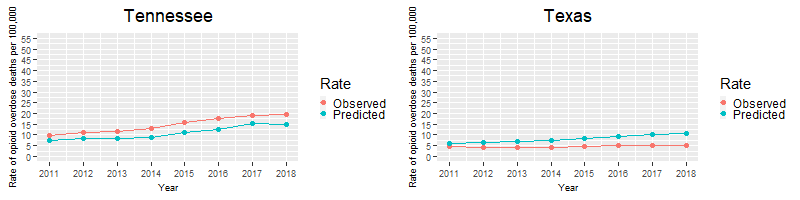

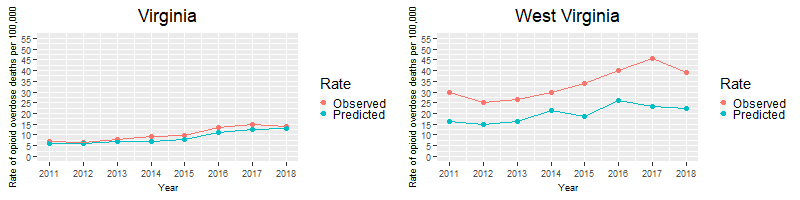


**Figure S5D**: Overdose mortality rates (Western States)


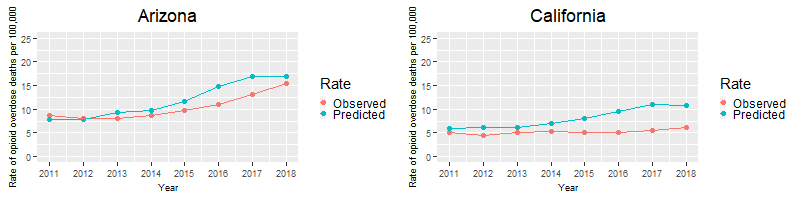

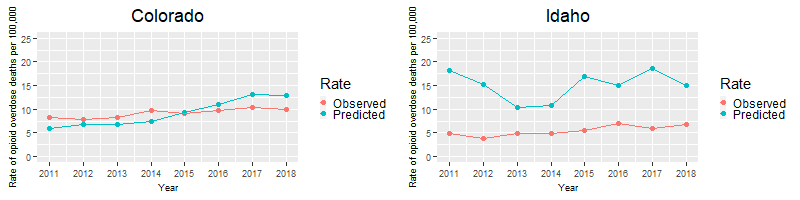

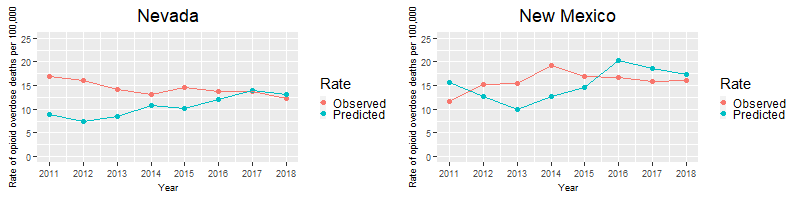

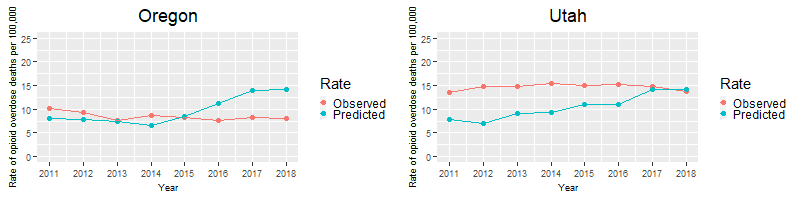

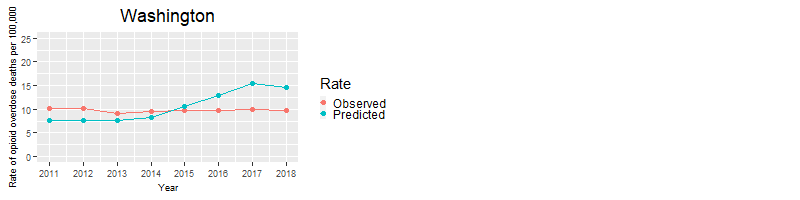

Supplement: Multimedia Appendix 1 [file jmir_v24i12e41527_app1.docx]
